# The first quarter of the C-terminal domain of Abelson regulates the WAVE regulatory complex and Enabled in axon guidance

**DOI:** 10.1186/s13064-020-00144-8

**Published:** 2020-05-02

**Authors:** Han Sian Joshua Cheong, Mark Nona, Samantha Barbara Guerra, Mark Francis VanBerkum

**Affiliations:** Department of Biological Sciences, Wayne State University, Detroit, MI 48202 USA

## Abstract

**Background:**

Abelson tyrosine kinase (Abl) plays a key role in axon guidance in linking guidance receptors to actin dynamics. The long C-terminal domain (CTD) of Drosophila Abl is important for this role, and previous work identified the ‘first quarter’ (1Q) of the CTD as essential. Here, we link the physical interactions of 1Q binding partners to Abl’s function in axon guidance.

**Methods:**

Protein binding partners of 1Q were identified by GST pulldown and mass spectrometry, and validated using axon guidance assays in the embryonic nerve cord and motoneurons. The role of 1Q was assessed genetically, utilizing a battery of *Abl* transgenes in combination with mutation or overexpression of the genes of pulled down proteins, and their partners in actin dynamics. The set of *Abl* transgenes had the following regions deleted: all of 1Q, each half of 1Q (‘eighths’, 1E and 2E) or a PxxP motif in 2E, which may bind SH3 domains.

**Results:**

GST pulldown identified Hem and Sra-1 as binding partners of 1Q, and our genetic analyses show that both proteins function with Abl in axon guidance, with Sra-1 likely interacting with 1Q. As Hem and Sra-1 are part of the actin-polymerizing WAVE regulatory complex (WRC), we extended our analyses to Abi and Trio, which interact with Abl and WRC members. Overall, the 1Q region (and especially 2E and its PxxP motif) are important for Abl’s ability to work with WRC in axon guidance. These areas are also important for Abl’s ability to function with the actin regulator Enabled. In comparison, 1E contributes to Abl function with the WRC at the midline, but less so with Enabled.

**Conclusions:**

The 1Q region, and especially the 2E region with its PxxP motif, links Abl with the WRC, its regulators Trio and Abi, and the actin regulator Ena. Removing 1E has specific effects suggesting it may help modulate Abl’s interaction with the WRC or Ena. Thus, the 1Q region of Abl plays a key role in regulating actin dynamics during axon guidance.

## Introduction

Abelson tyrosine kinase (Abl) is a key signaling molecule involved in a plethora of cell processes [1–4]. Arguably, it might be best known as a key regulator of actin dynamics during development of the organism, including cell morphogenesis, migration, and axon guidance in both vertebrate and invertebrate model systems [1–3]. Here, we focus on the function of the sole *Abl* homolog in *Drosophila* during development of axon tracts. Loss of maternal and zygotic *Abl* in the embryo results in disruption of epithelial morphogenesis, and also severely impacts commissure formation in the nerve cord [5, 6]. However, if maternal loading is retained, zygotic loss of *Ab*l results in more specific axon guidance defects, revealing key roles for Abl as a link between upstream guidance receptors and the cytoskeletal elements underlying growth cone extension and steering.

In the embryonic nerve cord, zygotic loss of *Abl* causes aberrant crossing of axons at the midline [7–10], a simple phenotype that has been helpful in genetic analyses linking Abl activity to the transduction of both midline attractive (Frazzled) [8, 11, 12] and repulsive (Roundabout) [10, 13, 14] cues. *Abl* and *Dscam1* also genetically interact in dendritic arborization [15]. In addition, loss of Abl signaling impairs motoneuron axon guidance in the intersegmental nerve b (ISNb) and segmental nerve a (SNa) [16, 17]. In modulating these axon guidance events, Abl cooperates with many partners known to be important in regulating cytoskeletal dynamics, such as the actin-regulating proteins Enabled (ena) [18, 19], Abelson-interacting protein (Abi) [20] and Chickadee/profilin [16], the adaptor protein Disabled [17], and the Rac guanine exchange factor (GEF) Trio [21]; even the microtubule-associated protein Orbit may be a target [14]. Indeed, Abl is thought to play a key role in balancing between linear and branched actin polymerization that underlie growth cone dynamics [21]. However, a well-defined, complete Abl signaling pathway remains elusive, due to the large number of Abl partners and likely interplay between them.

Initial work focused on the identification of Abl phosphorylation targets, most notably Roundabout, Frazzled, Ena, Abi and Trio [8, 13, 22, 23], although in some cases, the significance of phosphorylation remains in question (e.g. see [22]). Moreover, early experiments in Drosophila also demonstrate that a kinase inactive-version of Abl retains significant function, even rescuing lethality of *Abl* loss [24], although partial loss of function is demonstrated in developmental events in maternal-zygotic *Abl* mutant embryos [5]. Thus, even without kinase activity, Abl is still a key player in regulating actin dynamics, yet how Abl does so remains in question. To further develop our understanding of Abl signaling, we assessed the contribution of other regions of Abl to its function.

Early work demonstrated that the long C-terminal domain (CTD) of Abl is critical to its function, as the CTD of murine Abl1 cannot substitute for the Drosophila Abl CTD in vivo [24]. The CTD of Drosophila Abl spans ~ 1100 amino acids and contains no known domains except for a small F-actin binding domain (Fig. 2a). In addition, two putative EVH1-binding motifs (F/LPPPP) were initially thought to recruit Ena [25]. Yet both regions can be removed without unduly affecting Abl function in flies [5, 11]. Homology-based methods comparing insect Abl homologs alone identified 4 small conserved regions of the CTD, but 3 of the 4 can be removed with no apparent effect [5]. The sole important region (‘conserved region 1’, or CR1) contains a PxxP motif that may be functionally relevant [5], as discussed below.

On the other hand, the Abl CTD is characterized by the presence of intrinsic disorder spanning most of its sequence [26]. Intrinsically disordered regions lack well-defined secondary or tertiary structure, and are flexible and structurally heterogenous [27]. Yet, the role of intrinsically disordered regions in cell signaling is increasingly appreciated, due to their potential ability to bind multiple protein partners, as well as enrichment in post-translational modification sites [28]. Furthermore, disordered regions appear to have faster evolutionary rates [29, 30], perhaps explaining the limited homology between vertebrate and invertebrate species, as well as the limited success of homology-based approaches to understanding Abl CTD function. We reasoned that the long CTD of Abl is a prime candidate for a scaffolding function, thus accounting for its critical importance in *Drosophila* Abl function.

Our earlier study tested this hypothesis by systematically analyzing the contribution of the CTD region to Abl function in viability and axon guidance [26]. The CTD was somewhat arbitrarily divided into four large regions (we called them ‘quarters’, or 1Q-4Q), taking care not to disrupt the few known peptide motifs. These regions were then either deleted from full-length Abl or added onto the Abl N-terminus alone, and the resulting transgenes were tested in axon guidance. Surprisingly, this analysis revealed that much of the CTD could be removed without significantly altering Abl function in axon guidance or viability. Indeed, only one region, 1Q, was identified as absolutely necessary for Abl function, although another region, the third quarter, aided in localizing Abl to axons, allowing a small transgene carrying only 1Q and 3Q with the N-terminus (AblN-1Q-3Q) to behave close to a wild-type Abl.

Unfortunately, few sequence motifs may be found within the 1Q region. The second half of 1Q is similar to the CR1 region which plays a role in actin based morphogenetic events [5] and is slightly more conserved within insects. This region also harbors a small PxxP motif that may mediate physical interactions with SH3 domains, which are widely distributed among proteins in nature [31, 32]. In vertebrates, three such motifs mediate interactions with the SH3 domains of Abi, Crk, Nck, and Grb [33–38]. Thus, these proteins are potential candidates for binding to 1Q in *Drosophila*, although we note that the conservation of the surrounding amino acids, which often contribute to binding [39], are much poorer [26]. To dissect these regions, we divided the 1Q region into halves, calling them the first and second eighths (1E and 2E), and also created a third mutant removing only the four amino acids of the PxxP motif. Deletion of either 2E or the PxxP motif alone significantly inhibits Abl function in axon guidance [5, 26]. However, deletion of the entire 1Q sequence impacts Abl function even more, suggesting the presence of other unknown regions of interest [26].

Here, we continue to investigate why the 1Q region is critical to Abl function in axon guidance. First, we screened for protein binding partners of 1Q by GST pulldown, and identified Hematopoietic protein (Hem) and Specifically Rac1-associated protein 1 (Sra-1). Both proteins are part of the WAVE regulatory complex (WRC), a 5-subunit complex that is activated by Rac GTPases to nucleate branched actin polymerization [40–43]. Interestingly, the WRC component Abi, as well as the Rac GEF Trio, are known to genetically interact with Abl during axon guidance [8, 20, 21]. As such, we elected to continue genetic analysis of the Abl-Hem-Sra-1 interaction to determine if the 1Q region regulates the WRC through these other Abl partners. Genetic analyses indicate that 1Q, and especially 2E and its PxxP motif, does indeed work with the WRC in axon guidance, as mutations of these regions genetically interacts with *Abi* and *trio*. It has been proposed that the WRC acts in parallel to an Ena-dependent pathway [21], yet as an unanticipated outcome, the 1Q region is also important for Abl’s ability to regulate Ena despite the lack of any Ena-binding motifs in 1Q. Clearly, the 1Q region and especially 2E and its PxxP motif, connects Abl to multiple players of actin dynamics. On the other hand, the 1E region may be involved in fine-tuning Abl function with the WRC and Ena. We suggest that 1Q functions at the intersection between linear and branched actin polymerization, and aids Abl in linking receptors to modulate this balance during axon guidance.

## Methods

### Recombinant GST protein expression and purification

The Abl 1Q region was amplified from a pMT-Abl vector as an AgeI/NotI fragment and cloned into a modified pGEX-6p1 vector that adds a C-terminal FLAG tag. The forward and reverse primers used are TTG AAC CGG TCA *TGC GCT GGA GCA CAT GTT T* and GTG AGC GGC CGC *GTC CAT TCG TGC TGA GGT CGT C *(italicized regions are complementary to 1Q). Transgenic GST and GST-1Q were expressed in BL21 *E. coli* at 18 °C for 2 days in autoinduction medium [44]. *E. coli* was pelleted and lysed using B-PER reagent (Thermo Fisher, Waltham, MA, USA), and purified on Glutathione Sepharose 4B beads (GE Healthcare, Chicago, IL, USA). Following elution using 10 mM reduced glutathione + 1% Nonidet P-40, eluates were dialyzed against dialysis buffer (1% Nonidet P-40, 25 mM Tris pH 7.4, 150 mM NaCl). Protein concentrations were determined using Advanced Protein Assay reagent (Cytoskeleton, Inc., Denver, CO, USA).

### GST pulldown

Roughly 4 ml of frozen overnight collections of embryos were lysed in 20 mL total volume of lysis buffer (0.25% IGEPAL CA-630, 25 mM Tris pH 7.4, 100 mM NaCl, 5% glycerol, 1 mM MgCl_2_, 1 mM CaCl_2_, 10 mM NaF, 2 mM Na_3_VO_4_ pH 10, 1 mM ATP, 1x Problock Gold protease inhibitor cocktail (GoldBio, St Louis, MO, USA) by homogenization in a 15 mL Dounce homogenizer (10 strokes of each pestle), then sonicated for 1 min total sonication time with 5 s per pulse and 10s breaks at amplitude 5 with a Misonix S-4000 Sonicator (Qsonica, Newtown, CT, USA) with a 1/16″ probe. Debris was pelleted by centrifugation for 10 min at 7.2 k xG. Lysate was consecutively precleared for 20 min in 2 ml, 1 ml, and 0.5 mL of Glutathione Sepharose 4B beads (GE Healthcare, Chicago, IL, USA).

For pulldown, 75 uL Glutathione Sepharose 4B beads were loaded with 200 μg bait protein (GST, GST-1Q). Beads were incubated with 3 mL of lysate for 1.5 h at 4 °C, then washed 5 times with 500 uL lysis buffer. Co-purified proteins were eluted in 3 x 33uL 50 mM glutathione in lysis buffer (+ 0.3 M Tris pH 7.4), and fractions were combined. All experiments were performed in triplicate, with separate preparations of embryo lysate.

In preparation for mass spectrometry, polyacrylamide gel electrophoresis was carried out with either Mini-PROTEAN TGX 4–20% precast gels (Bio-Rad, Hercules, CA, USA) or hand-cast gels with a neutral-pH gel buffer (0.1 M tris-AcOH pH 7, 0.36 M Glycine). Gels were run with a running buffer of 25 mM tris, 192 mM glycine, 0.1% SDS. Whole protein staining with Sypro Ruby stain (Thermo Fisher, Waltham, MA, USA) was carried out as per manufacturer instructions, and regions of interest (matched areas between GST and GST-1Q, above and below bait bands) were excised for mass spectrometry.

### Mass spectrometry and analysis

32 gel slices were submitted to the Wayne State University Proteomics Core. The gel pieces were first washed with water and 25 mM NH_4_HCO_3_, 50% ACN for 15 min each. The liquid was removed, and the gel pieces were dehydrated in 100% ACN for 5 min, rehydrated in 50 mM NH_4_HCO_3_, followed by addition of an equal volume of 100% ACN. After incubation for 15 min, all liquid was removed and the gel pieces dehydrated once again in 100% ACN for 5 min, and vacuum-desiccated for 5 min in a speed vac. The following was then performed: reduction with 5 mM DTT, 50 mM NH_4_HCO_3_; alkylation with 15 mM IAA, 50 mM NH_4_HCO_3_; and overnight digestion with sequencing-grade trypsin (Promega, Madison, WI, USA) in 40 mM NH_4_HCO_3_, 0.01% Protease Max (Promega, Madison, WI, USA), and 1 mM CaCl_2_. Following digestion, peptides were extracted from the gel plugs using 0.5% TFA, desiccated and solubilized in 0.1% FA.

The peptides were separated by reversed-phase chromatography with Acclaim PepMap100 C18 columns (Thermo Fisher, Waltham, MA, USA), followed by ionization with the Nanospray Flex Ion Source (Thermo Fisher, Waltham, MA, USA), and introduced into a Q-Exactive versus Fusion mass spectrometer (Thermo Fisher, Waltham, MA, USA).

Abundant species were fragmented with high energy collision-induced dissociation (HCID for QEx) or collision-induced dissociation (CID for Fusion). Data analysis was performed using Proteome Discoverer 1.4 (Thermo Fisher, Waltham, MA, USA) which incorporated the Mascot (Matrix Science, Boston, MA, USA) and Sequest algorithms (Thermo Fisher, Waltham, MA, USA). The Uniprot_Dros_Compl_20160407 database was searched for *Drosophila* protein sequences and a reverse decoy protein database was run simultaneously for false discovery rate (FDR) determination. Secondary analysis was performed using Scaffold 4.5.3 (Proteome Software, Portland, OR, USA). Minimum protein identification probability was set at <=1–2% FDR with 2 unique peptides at <=1% FDR minimum peptide identification probability.

Mascot, Sequest, and X! Tandem were searched with a fragment ion mass tolerance of 0.02/0.05 Da and a parent ion tolerance of 10/20 PPM. Carbamidomethylation of cysteine was specified in Mascot, Sequest, and X! Tandem as a fixed modification. Deamidation of asparagine and glutamine, oxidation of methionine, and acetylation of the N-terminus were specified in Mascot & Sequest as variable modifications. Glu- > pyro-Glu of the n-terminus, ammonia-loss of the N-terminus, gln- > pyro-Glu of the N-terminus, deamidation of asparagine and glutamine, oxidation of methionine, and acetylation of the N-terminus were specified in X! Tandem as variable modifications.

### Fly genetics and stocks

*Drosophila melanogaster* were cultured at 25 °C on standard cornmeal-molasses medium on a 12 h light/dark cycle. The following alleles, deficiencies and drivers were used: *Abl*^*4*^*, Abl*^*2*^*, trio*^*1*^*, Abi*^*KO*^*, Hem*^*J4–48*^_,_*ena*^*23*^*, insc*^*Mz1407*^*(1407-Gal4)*, *elav-Gal4* (on chromosome 2)*,* and *Df(3R)Exel6174* (*Sra-1*^*Df*^). *UAS-Abl* transgene stocks are as described below. In addition, the following UAS transgenes were also used: *UAS-trio. B* (on chromosome 3) [45], *UAS-ena. His6* (on chromosome 3), *UAS-mCherry.Abi* (on chromosome 2 at ZH-51D attP landing site), and *Sra-1*^*EY06562*^ (*UAS-Sra-1*). Stocks were balanced over LacZ-containing balancer chromosomes (*TM3 Sb act-lacZ, CyO elav-lacZ, TM6 ase-lacZ* or *TM6 T8-lacZ*) where appropriate. *ena*^*23*^ stocks were instead balanced over the nearby *betaTub56D*^*k00705*^ LacZ enhancer trap due to stock health reasons. The *trio*^*1*^, *Abi*^*KO*^*,* and *UAS-trio122* stocks were a kind gift from Dr. Edward Giniger (NINDS). Other fly stocks were obtained from Bloomington Drosophila Stock Center or were pre-existing in our lab.

### Transgenic constructs

UAS-Abl transgenes that are wild-type or carry the Δ1Q, Δ2E or ΔP deletions are as previously described [26]. The AblΔ1E transgene was made by site-directed mutagenesis of a PUASTattB-Abl plasmid using the following primers (regions complementary to Abl italicized): AGG AGA CCG GT*C TCA CGC CGA ACG CCC AC* and GTG AGA CCG GTC TCC *TGC TTT TCC ACC GCT TCG G*. The PCR product was digested with AgeI and self-ligated. All Abl transgenes used here were inserted into the *ZH-attP-22A* landing via Phi C-31 transgenesis [46]. All fly germline transformations were carried out by Rainbow Transgenic Flies, Inc.

### Immunoblotting

To determine transgenic expression levels, *Abl* transgenes were expressed in third instar larvae with *1407-Gal4* at 25 °C. Separately, a *UAS-RFP-FLAG* transgene was expressed with *1407-Gal4* and *elav-Gal4* at 18 °C and 25 °C. For both experiments, 5 nerve cords per genotype were dissected and lysed in 200 μL SDS sample buffer, ran on SDS-PAGE gels, and transferred to PVDF membranes. Blots were incubated with 1:10000 Rat anti-FLAG L5 (Thermo Fisher, Cat# MA1–142-1MG, Waltham, MA, USA) and 1:500 Mouse anti-beta-tubulin E7 (DSHB, Cat# E7-s, Iowa City, IA, USA) in 0.5% nonfat milk in PBS-Tween (1x PBS, 0.1% Tween-20). After washing, blots were incubated with 1:20000 HRP-conjugated Goat anti-rat antibody (Jackson Immunoresearch, Cat# 112–035-003, West Grove, PA, USA) and 1:20000 HRP-conjugated goat anti-mouse antibody (Jackson Immunotech, Cat# 115–035-003, West Grove, PA, USA) in 0.5% nonfat milk in PBS-Tween. Chemiluminescent detection was carried out with Clarity Western ECL Substrate (Bio-Rad, Hercules, CA, USA). Dissections and western blots were repeated 3 times.

### Fly mating schemes

Crosses for ISNb bypass with endogenous Abl present use the same basic scheme, where the female carries the Gal4 driver (*1407-Gal4* or *elav-Gal4*) and any loss-of-function alleles balanced against a LacZ balancer, while the male carries all *Abl* transgenes and any other transgenes used. Crosses in *Abl*^*4/2*^ mutants were carried out with a similar scheme, where the female carries the *Gal4* driver, *Abl*^*4*^ allele and any other loss-of-function alleles, while the male carries all transgenes and the *Abl*^*2*^ allele. However, as we carried out these mating schemes, we observed that a small proportion of embryos that were heterozygous for either *Hem, Sra-1* or *trio* (without inheriting any balancers), but had a female parent with the TM6 balancer, had unexplained, highly-penetrant bypass phenotypes. This observation is similar to a previously observed TM6 balancer-induced maternal effect [47]. This effect was not observed when only the male parent carried the TM6 balancer. Thus, we reversed the parental sexes of all crosses that require TM6 balancers (*Sra-1*^*Df*^ and *Hem*^*j4–48*^ for the bypass assay, and *Sra-1*^*Df*^ and *trio*^*1*^ for *Abl*^*4/2*^ embryos).

### Embryo collection and immunohistochemistry

Embryos were collected by standard techniques as previously described [7]. Flies were allowed to deposit embryos overnight on apple juice agar in mini embryo collection cages (Genesee Scientific, San Diego, CA, USA). Collected embryos were dechorionated with 25% bleach, fixed in formaldehyde-saturated heptane for 45 min with gentle rocking. Vitelline membranes were cracked by vigorous shaking in methanol, followed by immediate rehydration in PBT (1x PBS, 0.1% Triton X-100). To mark embryos carrying LacZ transgenes, embryos were then incubated in X-gal staining solution (1x PBS, 2 mM MgCl_2_, 5 mM potassium ferrocyanide, 5 mM potassium ferricyanide, 8 μL mL^− 1^ 5% X-gal in dimethylformamide) for 1–12 h. Embryos were post-fixed in 4% formaldehyde in PBS for 10 min.

Embryos were incubated with mouse monoclonal antibody 1D4 supernatant (DSHB, Cat# 1D4 anti-Fas II-s, Iowa City, IA, USA) at 1:10 dilution, or concentrate (DSHB, Cat# 1D4 anti-Fas II-c, Iowa City, IA, USA) at 1:100 dilution in PBT + 0.5% nonfat milk overnight. Following washing, embryos were incubated with polyclonal HRP-conjugated goat anti-mouse antibody (Jackson Immunotech, Cat# 115–035-003, West Grove, PA, USA) for 4 h. Chromogenic detection was carried out by incubation of embryos in Stable DAB solution (Thermo Fisher, Waltham, MA, USA). Embryos were then washed with PBT and cleared in 70% glycerol. Imaging was carried out on a Leica DM5500B (Leica Microsystems, Buffalo Grove, IL, USA) microscope with differential interference contrast optics and an HCX PL APO 100x/1.40–0.70 OIL objective. Images were captured with a Leica DFC425C color camera using the LAS AF acquisition software.

### Embryonic phenotype quantification

Embryos were sorted by developmental stages as previously defined [48], and were counted by a trained scorer blinded to embryo genotypes. Scoring was done on a Leica DM5500B microscope (Leica Microsystems, Buffalo Grove, IL, USA) at 1000x magnification with brightfield and differential interference contrast optics. For midline crossover defects, whole mount embryos of stages 16–17 were analyzed. All abdominal and thoracic segments were evaluated for each embryo. Ectopic crossing overs were defined as Fas2-positive axon bundles crossing the midline and joining the Fas2-positive fascicles on the opposite side. Three replicates per genotype and ~ 30 embryos per replicate were counted. Data were pooled for experiments that used the same control crosses. No estimation of required sample size based on statistical power was carried out.

For ISNb defects, only stage 17 embryos were counted, and only abdominal segments 2–7 were evaluated as these have identical ISNb projections. For the bypass phenotype, full bypass was defined as complete failure of the ISNb to de-fasciculate from the ISN, with no visible axons leaving at the normal branch point. Partial bypass was defined as segments with visibly thinner ISNb branches, branching at a later point than expected, or branching followed by rejoining to the ISN. For the stop short phenotype, the last position of successful innervation was recorded per hemisegment, which may be at any of the muscle clefts 12/13 (wild-type), 6/13 or 6/7. Hemisegments with ISNb bypass could not be evaluated for stop shorts, and thus were excluded from stop short analysis. A variable number of hemisegments were counted per embryo due to variations in embryo positioning and occasional damage. As such, the n-value represents number of hemisegments, as previously defined [16]. For each experiment, three replicates per genotype and 60–100 hemisegments over ~ 10 embryos per replicate were counted. Data were pooled for experiments that used the same control crosses. No estimation of required sample size based on statistical power was carried out.

### Statistical analyses

For mass spectrometry data, statistical analysis was carried out using Scaffold Viewer version 4.4.8 (Proteome Software, Portland, OR, USA). Spectral counts for each protein were compared between 3 replicates of GST-1Q and GST by pairwise t-tests with Hochberg-Benjamini correction at a threshold of *p* ≤ 0.05.

All other statistical analyses were carried out in R version 3.6.1 (R Foundation, Vienna, Austria) with the multcomp [49], MASS [50], ggplot2 [51], car [52], DHARMa (http://florianhartig.github.io/DHARMa/), and emmeans (https://github.com/rvlenth/emmeans) packages. For counts of midline crossing over defects in *Abl*^*4/2*^ embryos, data were pooled for all datasets that use the same control crosses, and fitted to a negative binomial generalized linear model with crossovers per embryo as the response variable, and *Abl* transgene and other genotype (manipulation of *Abl* pathway genes) as the two predictor variables. The dispersion test [53–55] was used to determine overdispersion. Diagnostics were carried out with scaled residual plots using the DHARMa package. Post-hoc analysis was carried out by comparisons of the estimated marginal means [56] with the emmeans package, at a threshold of *p* ≤ 0.05 with the Holm method of *p*-value adjustment. A set of pre-planned treatment-vs-control comparisons were made as follows: for each *Abl* transgene, crossing overs were compared between the *Abl*^*4/2*^ condition alone and other genotypes (manipulation of *Abl* pathway genes). Furthermore, within each genotype (‘treatment group’), the no *Abl* transgene condition was compared to each *Abl* transgene. *P*-value adjustment was applied after pooling all comparisons made for the particular dataset.

For counts of ISNb defects (bypass or stop-short), the severity of the bypass phenotype for each embryonic hemisegment was rank-ordered from least to most severe as follows: wild-type, partial bypass and full bypass. Similarly, the severity of the stop-short phenotype was rank-ordered from least to most severe as follows: innervation of muscles 12/13 (wild-type), 6/13 and 6/7. Data were pooled for all datasets that use the same control crosses, and fitted to an ordered logit model with bypasses or stop shorts as the response variable, and *Abl* transgene and genotype (manipulation of Abl pathway genes) as the two predictor variables. The proportional odds assumption was tested using the Brant test [57]. Post-hoc analysis was carried out by comparisons of the estimated marginal means [56] at a threshold of *p* ≤ 0.05 with the Holm method of *p*-value adjustment. A pre-planned set of treatment-vs-control comparisons were made for each *Abl* transgene where the untreated genotype condition (no genetic elements other than *Abl* transgene and driver) was used as the controls (p-value adjustment was applied after pooling all comparisons made for the particular dataset). For the crosses of *1407-Gal4* to *UAS-Abl* transgenes, models were separately fitted for overexpression of *Abl* transgenes alone (including *AblΔ2E* and *AblΔP*), and for overexpression of *Abl* transgenes with manipulation of *Abl* pathway genes (without *AblΔ2E* and *AblΔP*). Fitting of separate models was necessary to prevent rank-deficiency.

## Results

### The 1Q region of the Abl C-terminal tail recruits the WRC

The ~ 1100 residue Abl CTD is critical for Abl function in axon guidance and actin dynamics. The 1Q region, a ~ 150 residue region in the CTD situated directly after the kinase domain (residues 656–799 in isoform RF), is the only region absolutely essential for this function [26]. 1Q may play a role in protein-protein interactions, as it has intrinsic disorder that is predicted to become ordered upon protein binding [26] and has a PxxP motif which may bind SH3 domains [5, 26]. To examine this hypothesis, we carried out a pulldown with recombinant 1Q fused to glutathione S-transferase (GST-1Q) to isolate 1Q binding partners from *Drosophila* embryo lysate. Following elution, we separated proteins by SDS-PAGE, excised matched gel fragments in GST (control) and GST-1Q lanes, and identified enriched proteins by liquid chromatography-tandem mass spectrometry (LC-MS/MS). All pulldowns were carried out in triplicate.

**Fig. 1:**
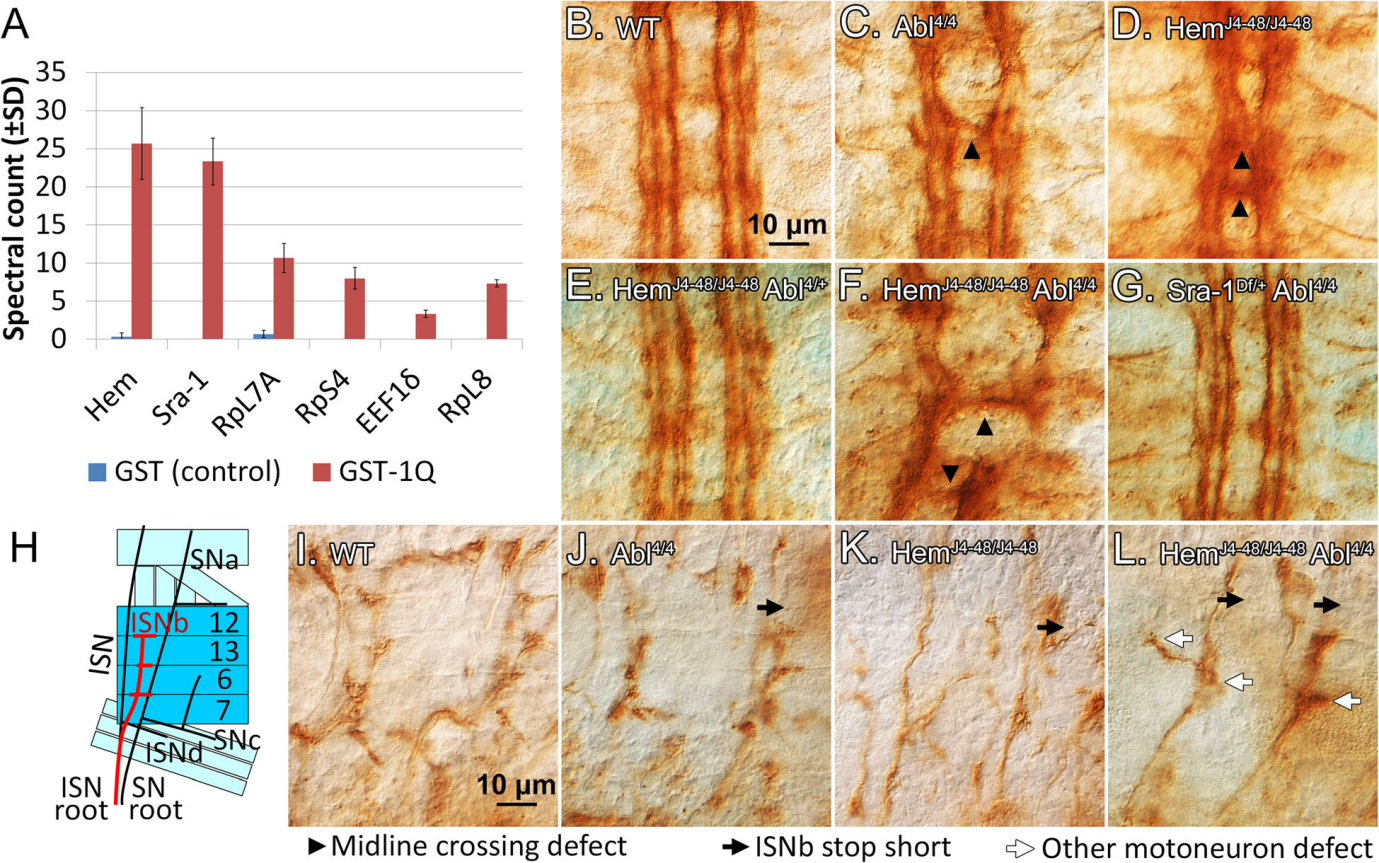
Hem and Sra-1 bind the first quarter (1Q) of Abl and genetically interact with Abl. **a** GST-1Q pulldown from *Drosophila* embryonic lysate, showing spectral counts of significantly enriched proteins from 3 replicates from mass spectrometry analysis. **b-g***Hem* and *Sra-1* genetically interact with *Abl* at the midline. **b** Wild-type embryo stained with mAb 1D4. The **c***Abl* and **d***Hem* homozygotes show crossing over defects (black arrowheads) at the midline. **e** Heterozygosity for *Abl* rescues *Hem* homozygotes, while **g** heterozygosity for *Sra-1* rescues Abl homozygotes. **f** The *Hem Abl* double homozygote is severely perturbed, with major defects in projections of both commissural and longitudinal axons. **h** Schematic diagram for motoneurons of abdominal segments A2-A7. The midline is at the bottom, and anterior is to the left. Note innervations of the ISNb (red) with muscles 7, 6, 13, and 12. **i-l***Hem* and *Abl* genetically interact in motoneuron axon guidance. (I) Wild-type ISNb projection in abdominal hemisegments A2–7. **j***Abl* and **k***Hem* homozygotes occasionally have stop short defects where the ISNb fails to innervate muscles 12/13 (black arrows). **l***Hem Abl* homozygotes have a severe increase in stop short defects and other axon guidance defects (white arrows). Pictures are representative of 3 sets of embryo collections

Mass spectrometry analysis detected 197 proteins in our pulldowns, with six proteins significantly enriched in the GST-1Q pulldown (Fig. 1a). The full data is found in Table S1 and Mendeley Data at https://data.mendeley.com/datasets/mw478mgmzs/1 [58]. Strikingly, the proteins Hematopoietic protein/Kette (Hem) and Specifically Rac-associated protein 1 (Sra-1) are the top two hits from the pulldown, and were present in approximately equal amounts at ~ 26 and ~ 23 peptide counts each (Fig. 1a). Hem and Sra-1 are well-known components of the actin-associated WAVE regulatory complex (WRC), along with subunits HSPC300, Scar/WAVE and Abelson-interacting protein (Abi) [59]. Neither Abi, a known Abl target, nor the other 2 WRC subunits were found in our pulldown. The remaining 4 proteins enriched in our pulldown consisted of ribosomal and ribosome-associated proteins, which are likely of lesser interest.

Interestingly, Hem and Sra-1 can form a heterodimeric subcomplex that can be separated from the rest of the WRC [60]. This pulldown suggests that the Hem/Sra-1 subcomplex may interact directly with 1Q. To establish the in vivo relevance of the putative Abl 1Q interaction with Hem and Sra-1, we elected to first use a genetic approach asking if these genes cooperate in the formation of both motoneuron projections and midline guidance.

### Hem and Sra-1 interact genetically with Abl in axon guidance

Embryonic axon tracts were visualized by immunohistochemistry with mAb 1D4 (anti-Fas2), which labels three longitudinal fascicles in the nerve cord, and motoneurons that project into the periphery. In late-stage embryos (stage 16–17 as previously defined [61]), the longitudinal fascicles do not cross the midline (Fig. 1b). Embryos heterozygous for *Hem*, *Sra-1* or *Abl* are wild-type, as are double heterozygote combinations. Embryos homozygous for *Hem* or *Abl* have occasional axon bundles that inappropriately cross the midline (Fig. 1c, d). The midline defects in *Hem* homozygous embryos, however, appear qualitatively different from *Abl* midline defects, compounding our ability to quantify these differences. Basically, *Hem* crossing overs are intermingled with narrowing of the nerve cord and thinning of fascicles, while *Abl* crossing overs appear as distinct bundles of axons. Strikingly, homozygous loss of both *Hem* and *Abl* severely enhances midline defects, as fascicles become highly disrupted, and axons at the commissures often fail to cross or cross inappropriately (Fig. 1f). Interestingly, in the homozygous/heterozygous combinations, heterozygosity of *Abl* rescues *Hem* loss, although heterozygosity of *Hem* does not in turn rescue *Abl* loss (Fig. 1e). Below, in the context of evaluating our 1Q mutants, midline crossing overs are counted for heterozygosity of *Hem* or *Sra-1* in *Abl* homozygotes in Fig. 4 and Tables S4–5.

These results are mirrored in the motoneurons at the periphery, where embryos homozygous for *Hem* or *Abl* have occasional defects in motoneurons (Fig. 1h-l, also see Fig. 3) However, motoneuron projections in *Hem Abl* double homozygotes are grossly abnormal with axons frequently misrouted or failing to extend (Fig. 1l). Clearly, *Hem* and *Abl* both play important roles in CNS and motoneuron axon guidance, although the severity of the double homozygous phenotype makes it difficult to draw more specific conclusions.

For *Sra-1*, we used a deletion that removes the *Sra-1* gene as well as several neighboring genes (*Df(3R)Exel6174*, hereby *Sra-1*^*Df*^). Given this limitation, we only used this mutation in its heterozygous state. Importantly, heterozygosity for *Sra-1* has no effect on development, but significantly suppresses the midline crossing defects observed in *Abl* mutant embryos (Fig. 1g). These data confirm the importance of Abl, Hem and Sra-1 in cooperating with each other during formation of these axon tracts. Although genetic suppression may occur whether Hem/Sra-1 and Abl function in parallel or within the same pathway, the physical interaction observed in our pulldown points towards the latter explanation. If so, we predict that this genetic interaction would depend on the 1Q region, and sought to test this idea using a known gain-of-function phenotype of *Abl*.

### Hem and Sra-1 cooperate with Abl in motoneuron axon guidance

**Fig. 2:**
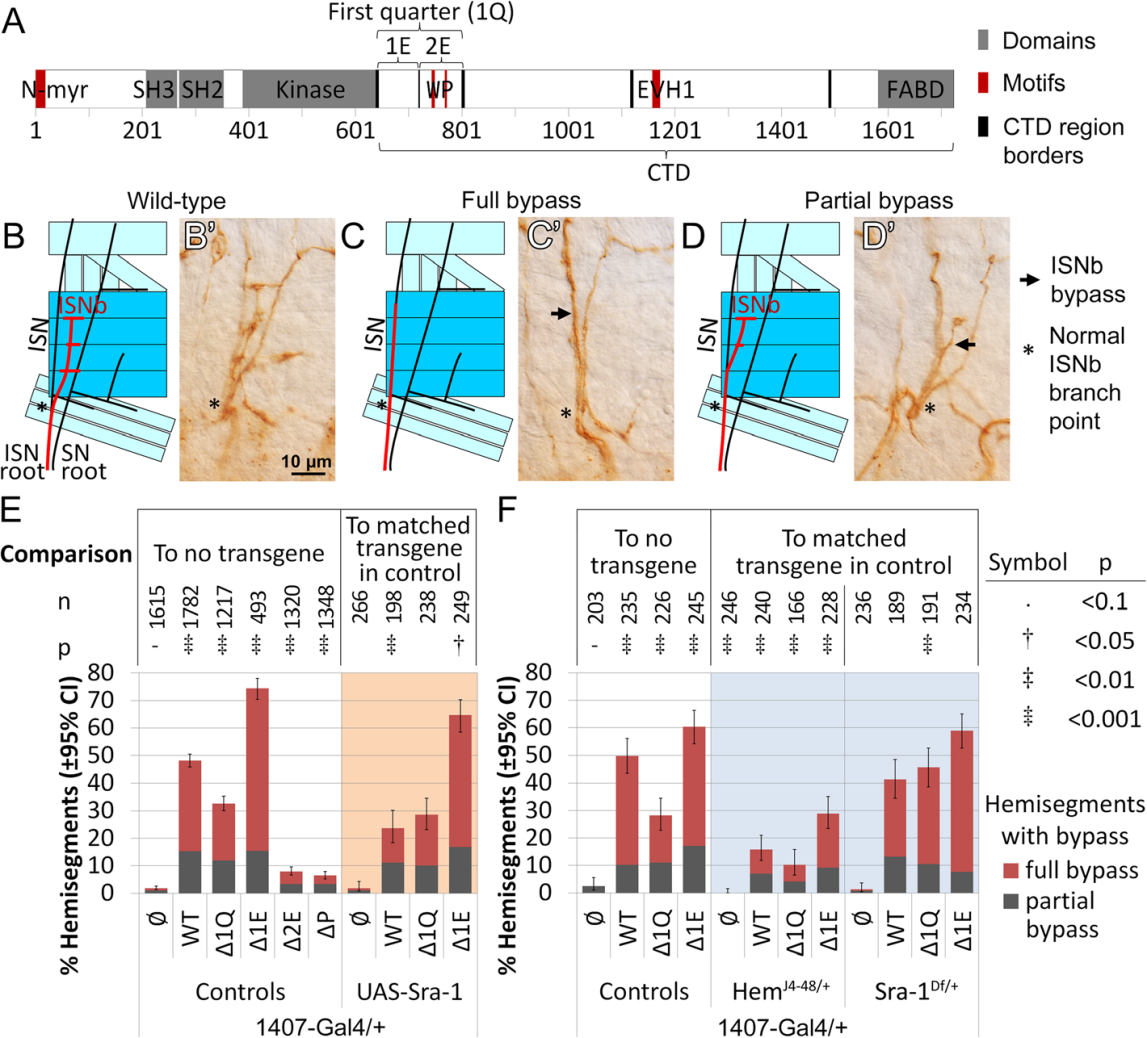
*Hem* and *Sra-1* modify an *Abl* gain-of-function ISNb bypass phenotype. **a** Diagram of Abl, showing domains in grey, and WIRS (W), PxxP (P) and EVH1-binding (EVH1) motifs in red. **b & b’** The ISNb (red) defasciculates from the intersegmental nerve (ISN) at a choice point (*). Overexpression of Abl causes ISNb bypass defects. **c & c’** In a full bypass, the ISNb (arrow) fails to defasciculate from the ISN, while **d & d’** in a partial bypass, the ISNb (arrow) defasciculates at a point more distal than expected. **e** ISNb bypass counts for *Abl* transgenes (WT, Δ1Q, Δ1E or no transgene, ∅) expressed with *1407-Gal4*, and interaction of these transgenes with gain of *Sra-1.***f** Bypass counts for interaction of *Abl* transgenes expressed with *1407-Gal4* with heterozygous loss of *Hem* or *Sra-1*. All counts are of individual hemisegments from 10 or more embryos, repeated over 3 or more sets of embryo collections; the n-value represents total hemisegments counted. Confidence intervals given are for penetrance of the phenotype. The direction of crosses is reversed between **e** and **f** due to a balancer-induced maternal effect (see methods)

In the abdominal segments A2 to A7 [62], motoneuron axon guidance in the intersegmental nerve b (ISNb) is sensitive to both loss and gain in Abl levels [63]. Overexpression of wild-type *Abl* in all neurons using the *insc*^*Mz1407*^ (*1407-Gal4*) driver causes a ‘bypass’ phenotype in the ISNb in about half of hemisegments, where the ISNb fails to defasciculate from the intersegmental nerve (full bypass) or separates at a further point (partial bypass, Fig. 2b-d) [63]. The ability of wild-type Abl to induce these guidance defects allowed us to first ask which of a battery of *Abl* transgenes deleting all or part of 1Q recapitulates the bypass phenotype. Briefly, the deletions are as follows: entire first quarter (Δ1Q), first eighth (Δ1E), second eighth (Δ2E) and PxxP motif (ΔP; see Fig. 2a for delineation of regions). These transgenes are inserted into the same Phi-C31 integrase site (ZH-22A) and are expressed at a similar level (Fig. S1 A) [26].

Only wild-type *Abl, AblΔ1Q* and *AblΔ1E* are capable of causing bypass, at 48, 33 and 74% of hemisegments, respectively, while deletion of 2E or PxxP greatly reduces bypasses to < 10%. These are intriguing data pointing to a major but complex role for 1Q. That is, bypass appears to require signaling from the 2E region (including the PxxP motif) but 1E influences its use: removal of only 1E greatly enhances bypass, while removal of the whole 1Q region lowers the efficacy of Abl to induce bypass. Might this reflect a role for the 1Q region in interacting with Hem or Sra-1? If so, our 1Q transgenes are predicted to be selectively sensitive to alterations in the dosage of either Hem or Sra-1 (overexpression or heterozygous loss). Importantly, neither homozygous loss nor overexpression of *Hem* or *Sra-1* are known to cause ISNb bypass on their own [64]. Thus, the ability of gain or loss of these proteins to alter ISNb bypass levels would likely indicate a specific role with Abl, perhaps through 1Q.

In this assay, we genetically perturbed *Hem* and *Sra-1* while overexpressing wild-type *Abl*, *AblΔ1Q* and *AblΔ1E*, as only these proteins cause significant bypass. In these crosses, the female flies initially carried all loss-of-function mutations. However, as we observed unexpected axon guidance defects, likely from a TM6 balancer-induced maternal effect [48], we used the reverse direction with heterozygous loss of *Sra-1* or *Hem* (and later *trio*) as explained in methods.

A heterozygous loss of *Hem* significantly suppresses the bypass phenotype for all 3 transgenes: from 50 to 16% for wild-type *Abl*, from 28 to 10% for *AblΔ1Q*, and from 60 to 29% for *AblΔ1E* (Fig. 2f). Thus, while the levels of *Hem* are important for Abl to generate a bypass, we could not detect a specific requirement for the 1Q region, The function of Hem in the WRC may be structural [59], suggesting that a partial loss of Hem leads to overall destabilization of the complex, and perhaps a general reduction in the WRC’s actin polymerization activity.

On the other hand, the effect of both gain and loss of *Sra-1* is selectively sensitive to alterations in the 1Q region. Overexpression of *Sra-1* reduces the ability of wild-type *Abl* to cause bypass (from 48 to 24%) as well as *AblΔ1E*, albeit less well (from 74 to 64%). However, *AblΔ1Q* continues to cause bypass at similar levels (from 33 to 28%, Fig. 2e). In support, removing half of Sra-1 does not change bypass for wild-type *Abl* (from 50 to 41%) and *AblΔ1E* (from 60 to 58%), but enhances bypass with expression of *AblΔ1Q* (from 28 to 46%, Fig. 2f). These data suggest that the 1Q region aids in Abl’s ability to function with Sra-1, although the genetic interactions do not necessarily support a simple activation/repression mechanism.

Given that Hem and Sra-1 are tightly linked in a heterodimeric subcomplex [59, 60, 65], yet only Sra-1 mutations exhibit sensitivity to mutations in 1Q,  we suspect that Sra-1 interacts with the 1Q region and brought Hem with it in our pulldown assay. This remains to be determined at a biochemical level, but interestingly, the 2E region includes a PxxP motif which may be recognized by the Abi subunit of the WRC [36, 37], and a putative WRC interacting receptor sequence (WIRS) motif [66] which may bind to a Sra-1/Abi dimer (Fig. 2a). If Abl binds to the Sra-1/Abi interface, it would be in an ideal location to modulate WRC activity, as Sra-1 plays a key role in regulating the WRC’s actin polymerization activity through Scar [59], and may be modulated or activated by two Abl partners, Abi and Trio (through Rac) [66, 67].

Although we did not detect Abi or Trio in our pulldown, it seemed likely that Abi and perhaps Trio may participate in Abl’s regulation of the WRC, a hypothesis we set out to test in loss of function *Abl* mutants. Zygotic loss of *Abl* results in a significant reduction in the levels of endogenous Abl, leading to well-documented defects in motoneuron projections and midline guidance. If Abl interacts with Abi and/or Trio to regulate WRC (via Sra-1), altering the dose of these target genes are expected to alter axon guidance and interact with our *Abl* transgenes in a 1Q-dependent fashion.

### 1Q is required for Abl regulation of WRC during ISNb extension

**Fig. 3:**
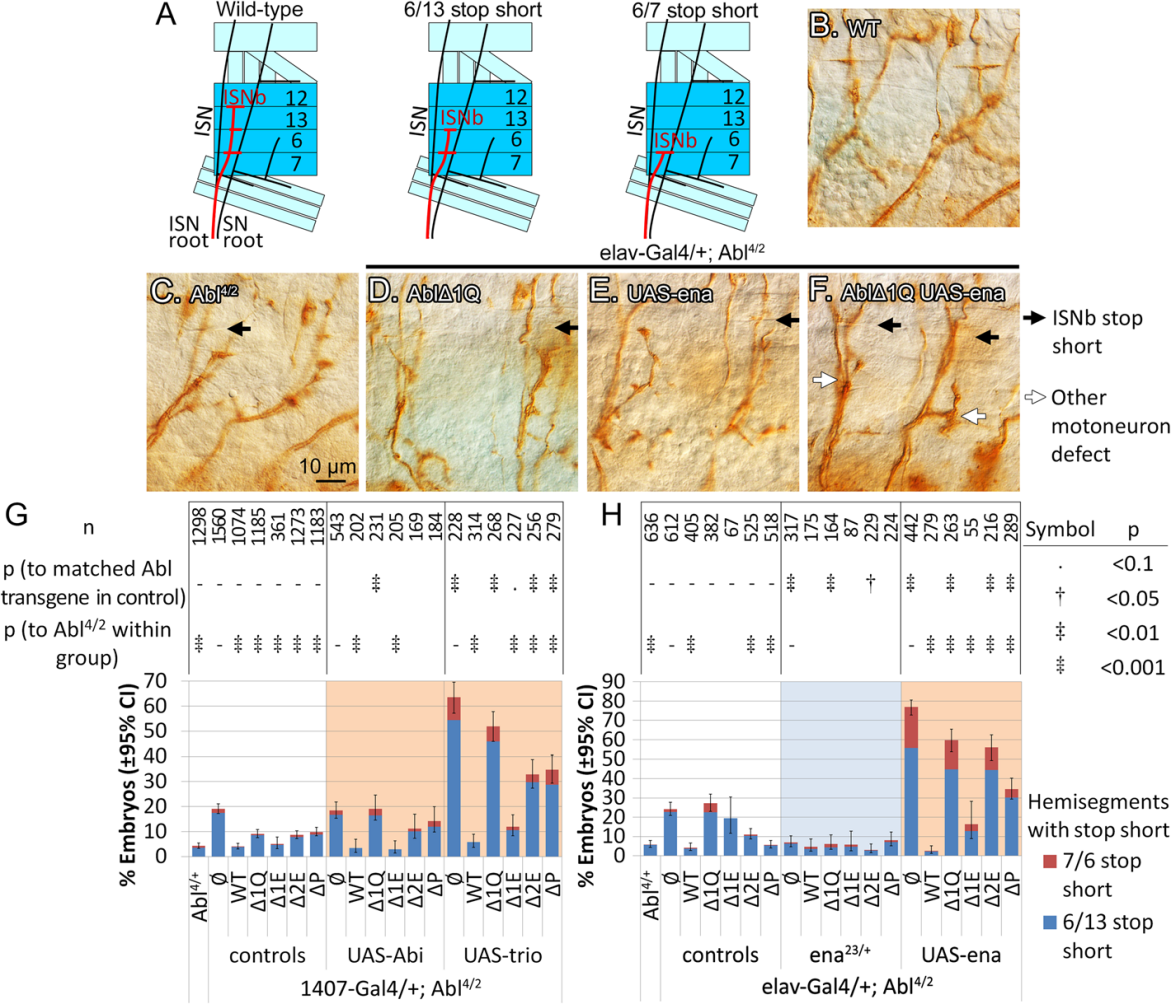
The 1Q region of Abl is required for Abi and Trio genes to modify ISNb stop shorts in *Abl* loss-of-function mutant embryos. **a** The ISNb innervates the clefts between muscles 6/7, 6/13 and 12/13. In *Abl* mutants, the ISNb occasionally fails to innervate the 12/13 cleft (6/13 stop short) or 6/12 cleft (6/7 stop short). **b** Wild-type ISNb innervations. **d***Abl* mutant showing ISNb stop shorts (black arrow indicates 12/13 cleft). Expressing either **d***AblΔ1Q* or **e***ena* increases stop shorts in *Abl* mutants. **f** Expression of both does not further increase stop shorts but causes variable misrouting or fasciculation defects in motoneuron projections (white arrows). **g** Stop short counts in *Abl* mutants expressing *Abl* transgenes, with overexpression of *Abi* or *trio* with *1407-Gal4*. **h** Stop short counts with heterozygous loss and overexpression of *ena* with *elav-Gal4*. All counts are of individual hemisegments from 10 or more embryos, repeated over 3 or more sets of embryo collections; the n-value represents total hemisegments counted. Confidence intervals given are for penetrance of the phenotype

Homozygous loss of *Abl* causes defects in axon guidance in the CNS and motoneurons, including ‘stop short’ of the ISNb [16]. In abdominal segments A2-A7 of late-stage embryos, the ISNb innervates 3 clefts between the muscles 6, 7, 12 and 13 (Fig. 3a, b). In the stop short phenotype, the ISNb fails to reach the muscle 12/13 cleft (6/13 stop short), or more rarely, the muscle 6/13 cleft (7/6 stop short) [16]. Loss of WRC components including Sra-1 [68], Abi and Scar [21] are known to cause stop short defects, suggesting that branched actin polymerization from WRC activity is necessary for ISNb extension. If so, might the Abl 1Q region modulate Sra-1 and its partners in this context?

In our hands, the *Abl*^*4/2*^ null condition causes ‘stop short’ defects in ~ 20% of hemisegments (Fig. 3g), close to previous reports using this allelic combination [16]. When expressed with *1407-Gal4*, stop shorts are rescued by a wild-type *Abl* transgene (from 19 to 4%) as expected (Fig. 3g). In contrast, rescue is incomplete with deletion of 1Q, 2E or P (~ 10% for all); surprisingly, deletion of 1E rescues to a similar level as the wild-type transgene (to 5%). These data demonstrate that 1Q, and especially 2E and its PxxP, are important for our transgenes to rescue motoneuron projections. As such, we predicted that rescue of stop shorts would also be selectively sensitive to alterations in the dose of the WRC pathway genes *Hem, Sra-1, Abi* and *trio.*

Unfortunately, gain of Sra-1, as well as heterozygous loss of *trio, Hem* or *Abi* all have little effect on the ability of our *Abl* transgenes to rescue stop shorts (the majority fall within the 2.5–10% range), which may reflect in part the limited dynamic range of this assay (Table S2 & S3). In addition, due to poor stock health, the effect of a heterozygous loss of *Sra-1* could only be evaluated using wild-type *Abl* and *AblΔ1Q* transgenes. Heterozygous loss of *Sra-1* rescues stop shorts in *Abl*^*4/2*^ embryos (from 18 to 3%) and does not alter wild-type *Abl*’s ability to rescue; less but statistically significant rescue is observed when *AblΔ1Q* is present (from 20 to 11%). These results suggest that excess Sra-1 activity contributes to stop shorts in *Abl* mutants, and, consistent with our bypass results, without the 1Q region, Abl has difficulty regulating Sra-1.

We also observe a clear requirement for 1Q for Abl to regulate over-expressed *Abi* and *trio.* Overexpression of *Abi* does not alter stop short levels in the *Abl*^*4/2*^ embryos alone, nor does it alter the rescue seen with a wild-type *Abl* transgene or *AblΔ1E* (Fig. 3g). Yet, expression of *Abi* with *AblΔ1Q* nullifies the partial rescue seen with *AblΔ1Q* alone (from 9 to 19%), suggesting that AblΔ1Q is deficient in regulating Abi. In comparison, overexpression of *trio* alone greatly exacerbates stop shorts in *Abl*^*4/2*^ embryos (from 19 to 63%; Fig. 3g), but there is a robust suppression of these with expression of wild-type *Abl* or *AblΔ1E* (to < 15%). In contrast, *AblΔ1Q* fails to rescue the increased stop shorts caused by increased Trio, while *AblΔ2E* and *AblΔP* are partially impaired (~ 35%). Together these data indicate that 1Q (and especially 2E and its PxxP) are important for Abl to suppress the effects of excess Trio. Note that our observation that overexpression of *trio* exacerbates stop shorts in *Abl* mutants contrasts with an earlier report indicating rescue of stop shorts [21]; however, in our hands, the *UAS-trio* transgene used in the prior work also exacerbates *Abl* stop shorts (Table S3).

Putting these results together, the 1Q region and especially 2E and PxxP, is required for Abl to suppress, or at least tightly control, the activity of Sra-1, Abi and Trio. A failure to do so results in stop short defects, probably reflecting a change in the regulation of branched actin formation [69]. It is intriguing that the 1Q region was sufficient to pull down Sra-1 (and Hem), and aids in the regulation of Sra-1, Abi and Trio during motoneuron extension. Future biochemistry will need to evaluate the full nature of a potential complex, including the individual roles of Abl, Sra-1, and Abi (and indirectly, Trio). As described below, our analysis with Enabled (Ena) suggests that it, too, may need to be included.

### Abl regulates Ena through 1Q

While the WRC is thought to control branched actin formation, Ena may work in a parallel pathway to regulate linear actin polymerization [21]. Together, these pathways regulate growth cone extension in response to guidance cues. Ena is well established as a binding partner and phosphorylation target of Abl [19, 22]. Genetically, heterozygosity of *ena* suppresses the axon guidance defects from homozygous loss of *Abl* [18, 19]. There is evidence that this genetic suppression stems from ectopic localization of Ena when Abl is lost [70, 71], which can then be alleviated by heterozygosity of *ena*. Given that Ena is recruited to the SH3 domain of Abl [22], we predict that an interaction between Abl and Ena would not involve 1Q, leaving Ena properly localized. Put another way, our 1Q mutant transgenes should be insensitive to a gain or loss of *ena* in these embryos. Unexpectedly, Abl 1Q mutant transgenes also appear to be defective in functioning with Ena.

To test for Ena’s role, we needed to use the pan-neural *elav-Gal4* driver instead of *1407-Gal4*, as the *1407-Gal4* insertion is in close proximity to *ena*. The *elav-Gal4* driver expresses at roughly 2-fold higher than *1407-Gal4* (Fig. S1 B), and this slightly alters the rescue of stop shorts by *Abl* transgenes: from a baseline of 24% stop shorts in *Abl*^*4/2*^, *AblΔ1Q* and *AblΔ1E* now fully fails to rescue *Abl* loss, at 27 and 19% respectively (Fig. 3h). This difference compared to *1407-Gal4* suggests that loss of 1E renders the Abl transgene more sensitive to expression levels, perhaps pointing to a role of 1E in limiting or fine-tuning Abl function; we also observe this at the midline (see further below). Rescue with other transgenes remain similar to that observed using the *1407-Gal4* driver.

Consistent with previous genetic results at the midline [18, 19], removing one copy of *ena* suppresses ISNb stop shorts in *Abl*^*4/2*^ embryos (from 24 to 7%). Likewise, removing half of *ena* improves the level of stop shorts seen with *AblΔ1Q* and *AblΔ2E* (from 27 to 6% and from 11 to 3%), indicating that excess Ena activity still contributes to stop shorts when AblΔ1Q and AblΔ2E are present. In contrast, loss of *ena* does not alter the rescue observed by wild-type *Abl*, *AblΔ1E* and *AblΔP* (5–8% for all). Thus, at least AblΔ1Q and AblΔ2E have trouble regulating Ena.

Overexpression of *ena* confirms the importance of 1Q in regulating Ena (Fig. 3b-f, h). Like *trio* above, overexpression of *ena* greatly exacerbates stop shorts in *Abl*^*4/2*^ null embryos (from 24 to 77%), and this is essentially rescued by a wild-type *Abl* transgene (to 3%). Interestingly, *AblΔ1E* readily suppresses stop shorts induced by overexpressed Ena (from 77 to 15%) while *AblΔ2E* and *AblΔP* show a stepwise ability to rescue (from 77 to 56% and 35% respectively). This difference supports an important role for 2E and PxxP in regulating Ena. It is worth noting that the complete removal of both 1E and 2E, in our *AblΔ1Q* mutant, allows a slight decrease in stop shorts (from 77 to 60%), but also induces variable misrouting or fasciculation defects in other motoneuron branches (Fig. 3f). This data suggest that the 1E region plays a (undefined) role in determining how Abl interacts with Ena.

Both the gain and loss of *ena* data suggest that Abl requires the 1Q region (especially 2E and PxxP) to suppress Ena activity. This was unexpected as Abl is thought to bind to Ena via the SH3 domain, and possibly the EVH1-binding motifs in another part of its C-terminal domain. As Ena and Sra-1 are both regulated by the same 1Q region of Abl, we speculate that during these motoneuron guidance events, Abl regulates Ena via its role with the WRC (see discussion section). Given this surprising result, we elected to explore the role of 1Q during midline guidance, where the genetic interaction of *ena* and *Abl* has largely been examined [8, 10, 19]. The midline also allows us to test the role of 1Q in a different signaling context, as receptors upstream of Abl at the midline are likely not the same as those used in the ISNb [7, 8, 12, 13].

### Axon guidance at the midline also requires 1Q

Late-stage embryos stained with mAb 1D4 display three longitudinal fascicles in the nerve cord. In *Abl*^*4/2*^ homozygotes, roughly half of embryos show midline crossing over defects, where these fascicles will inappropriately cross the midline. As with ISNb stop shorts, we used the frequency of this phenotype to assess the function of our *Abl* mutant transgenes, and the role of 1Q with WRC proteins and Ena. As above, we predict that alterations in the 1Q region will be sensitive to gain or loss of these genes. Note that the penetrance of midline crossing overs is presented here as a proxy for the severity of the phenotype, but embryos may also display varying number of crossing overs (shown by color and accounted for in statistics); these will be noted when appropriate. The tabulated data is found in Tables S4–6.

**Fig. 4:**
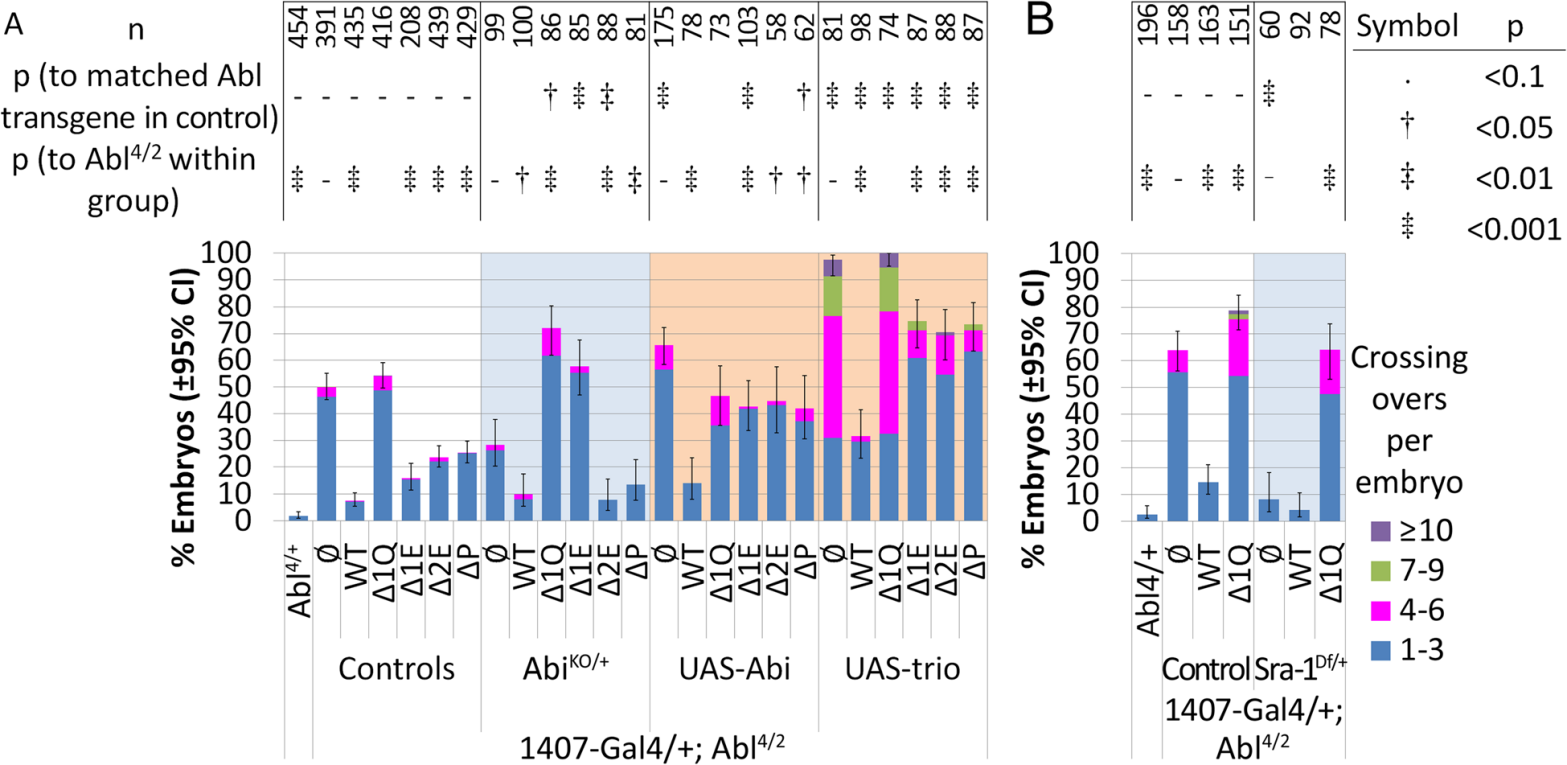
Abl 1Q regulates the WRC at the midline. **a** Midline crossing overs in *Abl* mutants expressing *Abl* transgenes with *1407-Gal4*, with gain or loss of *Abi*, or gain of *trio*. **b** Midline crossing overs in *Abl* mutants expressing *Abl* transgenes with *1407-Gal4*, with heterozygous loss of Sra-1. Confidence intervals given are for penetrance of the phenotype. All counts are from 3 or more sets of embryo collections, with ~ 30 embryos per set; the n-value represents embryo count. Confidence intervals given are for penetrance of the phenotype. The direction of crosses is reversed between **a** and **b** due to a balancer-induced maternal effect

In *Abl*^*4/2*^ mutant embryos, roughly 50% of embryos show midline crossing overs, at roughly 1–2 crossing overs per embryo. Abl function at the midline remains highly dependent on 1Q, especially 2E and its PxxP motif. With *1407-Gal4*, a wild-type *Abl* transgene rescues crossing overs to 8%, while *AblΔ1Q* fails to provide any rescue (54%; Fig. 4a). *AblΔ2E* and *AblΔP* only rescue about half as well (24 and 25% respectively), while *AblΔ1E* performs slightly better (16%). The need for 1Q and especially 2E regions to rescue crossovers suggests that Abl’s ability to function with the WRC is important in this guidance context. If so, the loss of 1Q, and especially 2E and its PxxP motif are predicted to be sensitive to changing the dosage of the WRC pathway genes *Hem, Sra-1, Abi* and *trio*. Our results align with this prediction, but we also observe a larger role of 1E.

With Sra-1, heterozygous loss of *Sra-1* greatly rescues the midline crossing overs of loss of *Abl* (from 64 to 8%, Fig. 4b), suggesting that excess Sra-1 activity contributes to these defects. Expression of *AblΔ1Q* completely prevents this rescue (to 64%) but other regions of 1Q could not be tested due to poor stock health. The converse effect is observed for gain of *Sra-1*, which slightly suppresses crossing overs with *AblΔ1Q* (from 50 to 40%), although genetic interactions with deletions of 1Q sub-regions were not significant (Table S4; see also for *Hem* data). Together, these data suggest that excess Sra-1 activity contributes to crossing overs, although the role of the regions of 1Q remains unresolved. On the other hand, both halves of 1Q appear to be important in regulating Abi and Trio at the midline.

Heterozygosity of *Abi* is reported to partially rescue crossing overs in *Abl* mutants [20]. Here, removing half of Abi levels in *Abl*^*4/2*^ embryos trends towards suppression (from 50 to 28%, Fig. 4a, S2), but this did not reach statistical significance. As expected, heterozygous loss of *Abi* has no effect on rescue by wild type Abl, while helping *AblΔ2E* (from 24 to 8%) and maybe *AblΔP* (from 25 to 14%). On the other hand, loss of Abi now completely prevents *AblΔ1E* from rescuing crossing overs (from 16 to 57%), and even enhances midline crossing overs for *AblΔ1Q* (from 55 to 72%). Interpreted directly, loss of 2E or PxxP seems to impair Abl’s ability to suppress Abi, but loss of 1E (in *Δ1E* and *Δ1Q*) instead reverses this effect. A clear molecular explanation for this result is not yet possible, but it does suggest an interplay between 1E and 2E regions in governing how this region works (see discussion). Conversely, overexpression of *Abi* alone in *Abl*^*4/2*^ embryos increases crossing overs from 50 to 66%, but expressing wild type *Abl* with *Abi* still rescues most crossovers (14%). On the other hand, when expressed with *Abi*, all 1Q mutant transgenes have difficulty bringing the over-expressed Abi under control—for Abl*Δ1E, Δ2E* and *ΔP,* overexpression of Abi increases embryos with crossing overs to 42–45%, while level of crossing overs remain high and unchanged for Abl*Δ1Q.* These results suggest that Abl inhibits Abi activity in order to prevent crossovers, and while 2E and PxxP are involved, the 1E region also exerts a regulatory effect.

Likewise, overexpressing *trio* in *Abl*^*4/2*^ mutants results in a large increase in the frequency and expressivity of midline crossovers, but these are brought under control by co-expressing wild type *Abl*. As observed with Abi, all of our 1Q mutant transgenes have difficulty bringing excess Trio under control, resulting in significant numbers of crossovers (Fig. 4a).

Clearly, the 1Q region is important for the function of Abl with the WRC during midline guidance events. Similar to that observed with motoneurons, 2E and PxxP remain the most important regions of 1Q to regulate both Abi and Trio during midline guidance. But at the midline, the 1E region seems to play a larger role, seemingly to influence how 2E communicates with these targets. This may reflect the specific axon guidance pathways utilizing Abl at the midline, or a greater need for precisely-regulated Abl activity for axons to navigate the midline.

### Ena regulation at the midline requires 1Q

Having established the requirement of 1Q and the WRC at the midline, we next turned to Ena. Heterozygous loss of *ena* suppresses midline crossing defects of *Abl* mutant embryos [8, 10, 19], suggesting that Abl inhibits Ena to facilitate midline crossing. Based on our motoneuron data, and contrary to our original expectations, the 1Q region, including 2E and its PxxP should be important for Abl to regulate Ena at the midline.

**Fig. 5:**
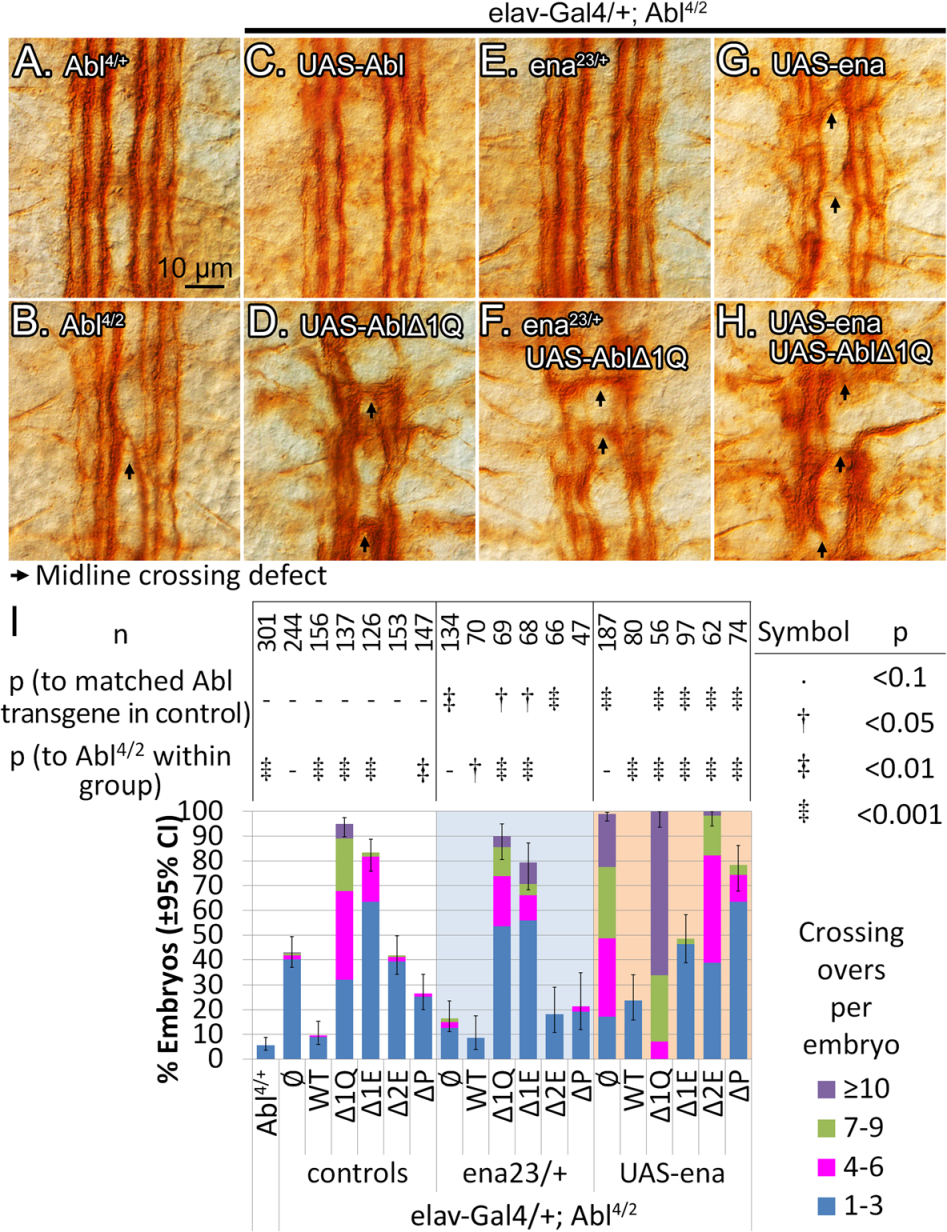
Abl 1Q is required to regulate Ena at the midline. **a-h** Late-stage embryonic nerve cords stained with mAb 1D4. **a** Wild-type embryo. **b***Abl* homozygotes show occasional midline crossing overs, rescued by expression of **c** wild-type *Abl* with *elav-Gal4*. **d** Expression of *AblΔ1Q* with *elav-Gal4* further increases crossing over. **e** Midline crossing overs are partially rescued by heterozygosity of *ena.***f** With *ena* heterozygosity, *AblΔ1Q* still shows many midline crossing overs. **g** Overexpression of *ena* further increase crossing overs, and **h** expression of *ena* behaves synergistically with *AblΔ1Q* to cause numerous defects. **i** Quantification of crossing overs in *Abl* mutants expressing *Abl* transgenes with *elav-Gal4*, with heterozygous loss or overexpression of *ena*. Confidence intervals given are for penetrance of the phenotype. All counts are from 3 or more sets of embryo collections, with ~ 30 embryos per set; the n-value represents embryo count. Confidence intervals given are for penetrance of the phenotype

Recall that for Ena, we had to use the *elav-Gal4* driver instead of *1407-Gal4*. Expression of *Abl* transgenes in *Abl*^*4/2*^ embryos with *elav-Gal4* (~ 2-fold stronger than *1407-Gal4*) reveals an expression level effect that highlights the importance of 1E. Counts for most *Abl* transgenes remain similar to *1407-Gal4* (Fig. 5i & Table S6), but here both *AblΔ1Q* and *AblΔ1E* now greatly exacerbate crossing overs to 95 and 83% respectively (Fig. 5d, i). This contrasts with *1407-Gal4* where *AblΔ1Q* does not further perturb *Abl*^*4/2*^ crossing overs, while *AblΔ1E* rescues them. We speculate that the increased sensitivity to the dose of transgene reflects the different signaling pathways converging on 1E to fully regulate Abl activity at the midline. Nevertheless, we could still evaluate whether our Abl transgenes selectively regulate Ena at the midline.

In *Abl* null embryos, heterozygous loss of *ena* rescues crossing overs from 43 to 16% (Fig. 5e, i), suggesting that excess Ena contributes to midline defects, as observed by others [19]. Removal of half of *ena* rescues crossing overs when *AblΔ2E* is expressed (from 48 to 18%), while *AblΔP* remains relatively strong in its ability to rescue (20%). On the other hand, removing half of Ena has only a small effect on the large number of crossing overs observed with *AblΔ1Q* or *AblΔ1E* expression, suggesting that these crossing overs may reflect more than a loss in Ena regulation. These results point to a role for 2E in suppressing Ena at the midline, a notion more clearly demonstrated when we overexpress Ena in Abl mutants.

Overexpression of *ena* in the *Abl*^*4/2*^ null greatly increases midline crossing overs, from 43 to 99%, although this is essentially reverted when a wild-type *Abl* transgene is re-expressed (Fig. 5g, i). Expression of *AblΔ1Q* completely fails to revert *ena* overexpression; penetrance remains high at 100% and crossing overs per embryo are further elevated (Fig. 5h, i). In comparison, *AblΔ2E* and *AblΔP* perform slightly better but are still clearly impaired: while the penetrance of crossing overs remains high at 100 and 78%, the number of crossing overs per embryo decreases compared to *ena* overexpression alone (Fig. 5i). Gain of *ena* clearly demonstrates a requirement for 1Q, and especially 2E and PxxP, in order for Abl to suppress Ena during midline guidance.

Surprisingly, *AblΔ1E* rescues about half of midline crossing overs (to 48%), and is clearly better than *AblΔ2E* and *AblΔP* in bringing Ena under control. This contrasts with observations with *Hem, Abi* and *trio* where all 3 of these *Abl* transgenes perform at about the same level. That is, at the midline, removal of 1E has little impact on Abl’s ability to regulate Ena, yet 1E is required to regulate the WRC genes, which also require 2E and P. Thus, as detailed in the discussion, we hypothesize that 1E plays a role in biasing how Abl works with Ena or the WRC.

## Discussion

Our pulldown and genetic analyses provide some of the first insight into why the 1Q region of Abl is so important for its function. 1Q physically interacts with Hem and Sra-1, likely forming a link between Abl and the WRC. Our detailed genetic analyses with multiple different axon guidance endpoints confirm the importance of 1Q in Abl’s ability to function with these proteins and their partners in axon guidance, and further point towards the 2E and its PxxP motif as the most critical regions for this role.

Overall, the regions of 1Q contribute the following functions to Abl’s role in axon guidance: The 2E region and its PxxP motif are a core requirement for Abl, with deletion of either region having similar deleterious impact (removal of PxxP tends to be slightly milder). *Abl* transgenes with either 2E or PxxP removed are impaired in their ability to rescue axon guidance phenotypes in *Abl* mutant embryos, and further lose the ability to cause the gain-of-function ISNb bypass phenotype. As we discuss further below, the 2E region and its PxxP motif are also prime candidates to mediate protein-protein interactions with the WRC. Removal of 1E, on the other hand, results in an *Abl* transgene that still partially or nearly fully rescues *Abl* loss, but rescue is now more sensitive to the level of *Abl* transgene expression (e.g. compare *1407-Gal4* vs *elav-Gal4* at the midline) and the guidance event examined. For example, in the gain-of-function bypass phenotype, overexpression of *AblΔ1E* suggests that this protein is ‘overactive’, as it causes more defects than a wild-type transgene. Yet at the midline, AblΔ1E does not seem to be hyperactive, as it clearly suppresses *ena* overexpression at the midline and is poorer at suppressing *trio* or *Abi* overexpression. Taken together, we hypothesize that 1E may control the target selectivity of Abl, biasing an interaction towards Ena over WRC, perhaps in the context of the specific guidance choice point being investigated.

The hallmark of the 2E region is a PxxP motif, which the vertebrate literature suggests multiple candidates for binding, notably Abi, Crk, Nck, and Grb [33–38]. Here, we coupled a GST pulldown from embryo lysate with mass spectrometry for protein identification to provide an unbiased sampling of 1Q binding partners. We identified Hem and Sra-1 in roughly equal amounts, consistent with their known association as a heterodimeric ‘subcomplex’ [59, 60]. Double homozygous embryos of *Abl* and *Hem* display numerous guidance defects in the nerve cord scaffold and motoneuron projections. This genetic interaction was not previously documented, although perhaps not surprising given the importance of both Abl and Hem in actin dynamics. These proteins may also have an antagonistic relationship. Firstly, heterozygous loss of *Sra-1* rescues the midline crossing defects of *Abl* mutants, and similarly heterozygous loss of *Abl* suppress crossovers observed in *Hem* mutants. As homozygous loss of either *Hem* or *Sra-1* alone has been reported to cause midline defects similar to loss of *Abl* [64], the apparent antagonistic relationship may not reflect a simple on/off switch. Instead, we speculate that Abl selectively controls the WRC either in response to upstream receptors and/or affects the WRC’s subcellular localization, as observed with another Abl partner, Ena [71, 72]. Over-expressing *Abl* in motoneurons further supports the involvement of Hem and Sra-1 in Abl function, and a role for 1Q. Heterozygous loss of *Hem* unilaterally suppresses the bypass phenotype caused by *Abl* transgenes, whether or not 1Q is present, perhaps doing so through its partner, Sra-1, or by dictating the overall level of the WRC. In contrast, the effect of loss- and gain-of-function *Sra-1* mutations on bypass is sensitive to alterations in 1Q, hinting that Sra-1 may play a more direct role with this region.

The role of 1Q, however, is likely not limited to Hem and Sra-1. Along with both proteins, Abi, Scar/WAVE and HSPC300 are also core members of the WRC. However, these 3 remaining members were not identified in our pulldown, which could reflect our pulldown conditions (medium stringency due to nonionic detergent and moderate salt concentrations to facilitate embryo lysis) or the labile nature of regulatory interactions, which may be difficult to detect in pulldowns. Of the remaining 3 WRC members, Abi is the best candidate for a direct function with 1Q, as Abi interacts with Abl during axon guidance, and is thought to play a modulatory role with the WRC [73, 74]. Moreover, the 2E region (the CR1 region of Rogers et al. [5]) contains the PxxP motif that may bind Abi, is conserved among invertebrates, and is important for Abl’s role in several actin-based processes [5]. We further identify a putative WIRS motif in 2E of Abl (Fig. 2a), which may interact with Sra-1 and Abi [66]. Indeed, 2E and its PxxP are both important for Abl to bring overexpressed Abi under control at the midline and in motoneurons. Interestingly, heterozygous loss of *Abi* shows unique genetic interactions with 1Q mutants at the midline—it exacerbates defects with *AblΔ1Q* and *Δ1E* while instead partially rescuing defects with *AblΔ2E*. This result cannot be explained by a simple activation/repression mechanism. We speculate that this instead reflects a role of Abi with 1E in our hypothesized Abl target selection function, perhaps in relation with Ena (also see further below). Finally, the 1Q region, through Trio, also appears to impact the function of Rac GTPase as an upstream activator of the WRC. Rac is considered a major regulator of the WRC by direct binding of active Rac to Sra-1, allowing Sra-1 to activate the actin polymerization activity of Scar/WAVE [59, 75]. This may be reflected in our results with the Trio GEF (an activator of Rac), which exacerbates midline and motoneuron defects when overexpressed in *Abl* mutants. We observe that Abl requires 2E and PxxP to bring the effects of overexpressed Trio under control.

Surprisingly, we also find that 1Q is required for Abl to regulate Ena, despite no indication that 1Q can interact directly with Ena. This finding is all the more surprising given that the putative Ena-binding motifs elsewhere in the CTD are dispensable for Abl function [5]. Like Trio and Abi, Abl requires 2E and its PxxP motif to regulate Ena; overexpressing *ena* in *Abl*^*4/2*^ mutants causes midline and motoneuron axon guidance defects that are poorly, if at all, rescued by *Abl* transgenes lacking 1Q, 2E or PxxP. Conversely, heterozygous loss of *ena* suppresses midline and motoneuron defects in *Abl*^*4/2*^ mutant embryos expressing Abl lacking 1Q, 2E or PxxP to varying degrees, suggesting that misregulation or failure to suppress Ena contributes to the generation of these defects. Interestingly, others have demonstrated that Ena and Abi can interact directly [76], and this interaction plays a non-cell autonomous role in axon guidance of photoreceptor neurons. These authors also suggested that Abl was a candidate in regulating the Abi-Ena interaction. Our results are consistent with this idea, as both 2E and PxxP are apparently required to regulate Ena and Abi. Thus, while it remains possible that Ena and the WRC function in parallel downstream of Abl, our data suggests that both pathways are tightly linked by Abl and that this interaction requires a functional 1Q region. Clearly, future work with genetic analyses manipulating all 3 genes, as well as in vitro biochemistry will be needed to verify this potential regulatory triad.

How might our findings fit in with current ideas on motility and axon guidance? Fundamentally, cell migration is driven by the dynamics of actin-rich protrusions at the cell or growth cone periphery that includes balancing the rates of actin polymerization between branched and linear elongation of F-actin filaments [77]. Importantly, the activity of Arp2/3—the main target of the WRC—is thought to be the major driver of branched F-actin nucleation in lamellipodia [78], while Ena appears to be a linear actin elongation factor [79]. The relative amounts of branched nucleation and linear actin elongation determines the ratio of F-actin branches per length of filament, which in turn controls the speed and directional persistence of actin protrusions [77]. In this model, high Arp2/3 activity should favor directional persistence of lamellipodia, while high Ena activity may favor faster, more dynamic lamellipodia [80]. Based on this framework, Abl’s ability to regulate both the WRC and Ena places it in a key position to modulate lamellipodial dynamics, and thus, growth cone steering. This is certainly consistent with the multitude of axon guidance pathways that co-opt Abl as a downstream effector. Moreover, recent work has begun to characterize the contribution of Abl to growth cone morphology and fluctuations in actin dynamics at a biophysical level [81, 82].

Abl’s proposed ability to regulate growth cone steering through actin dynamics helps explain the guidance defects we observe in our assays. The Roundabout (Robo) receptor is likely the most well-studied receptor with respect to Abl signaling, and governs growth cone repulsion at the midline in *Drosophila*. Currently, Robo is thought to recruit Abl by direct binding, and Abl appears to be a negative regulator of Robo, perhaps via phosphorylation [13]. Yet, Robo also recruits Ena directly, and genetic analyses suggest that Robo’s activity in midline repulsion occurs partially through Ena [13]. Thus, Ena and Abl appear to work in opposite directions downstream of Robo. Our results agree with this, as we observe that Abl counters Ena activity in midline crossing through 1Q. In addition, Robo also activates Rac signaling through the Rac GEF Son of Sevenless [83], and in *C. elegans* at least, Robo likely regulates the WRC through Rac during axon guidance [84]. Thus, Abl, Ena and the WRC are implicated downstream of Robo. While Robo signaling likely involves direct regulation of each protein, the final outcome is probably fine-tuned by 3-way inter-regulation between Abl, WRC and Ena, a process that we predict will require the 1Q region of Abl. Recent work with vertebrate Robo also suggests a temporal component in Robo signaling - upon initial stimulation by the Slit ligand, Robo induces a transient outgrowth of filopodia through Ena, following which growth cone collapse occurs [85]. It is tempting to speculate that Abl is coordinating aspects of this regulation through its ability to balance the activity of Ena and the WRC. In the periphery, Abl signaling in the ISNb neurons also appears to involve the same fine tuning of WRC and Ena partners interacting with 1Q. However, less is known about the specific upstream partners of Abl in ISNb outgrowth, but the semaphorin-plexin pathway may be a good candidate for future study. Both Sema1a and Plexin-A mutants display ISNb and SNa stop short phenotypes that are very similar to that of Abl [86, 87]. Moreover, both vertebrate Abl and Ena have been identified as components of the Sema6A and Sema6D reverse signaling pathway [88, 89], and in *Drosophila*, Sema1a contains a putative Ena-binding site that is implicated in synaptogenesis [90]. Finally, as Sema1a signaling promotes midline crossing [91], the potential role of *Drosophila* Abl downstream of Semaphorin-plexin signaling may provide an ideal model to understand how upstream receptors modify Abl signaling to direct a growing axon.

## Conclusion

The 1Q region of Abl provides a physical link to two key components of the WRC, Hem and Sra-1, and is important in helping to regulate WRC activity during motoneuron outgrowth and midline guidance. The second half (2E) of 1Q and its PxxP motif are particularly important, as their removal alters the ability of Abl to function with Abi and Trio to regulate WRC activity, but surprisingly this same region is required for Abl to regulate Ena during axon guidance. Given that Abi and Ena interact to affect axon outgrowth of photoreceptors [76], we suggest that Abl, Abi and Ena may form an important regulatory triad, which can be tested in future work. Removal of the first half of 1Q (1E) has less distinct effects, but overall it seems this region is a likely candidate for fine tuning how Abl regulates the WRC and Ena during the formation of the different axon pathways. Together, this work extends our knowledge of how Abl participates in the regulation of both branched and linear actin dynamics, and our battery of mutants will be useful tools in future work to dissect this pathway, in particular in linking actin dynamics to upstream receptors.

## Supplementary information

**Additional file 1 Figure S1.** Expression levels of *Abl* transgenes and *Gal4* drivers. (A) Expression levels of *Abl* transgenes, as assessed by western blots from 3rd instar CNS expressing transgenes with *1407-Gal4*. (B) Comparison of expression levels of *1407-Gal4* and *elav-Gal4* at 2 temperatures. The drivers were used to drive expression of UAS-RFP in the CNS of 3rd instar larvae. All immunoblotting was carried out against the C-terminal FLAG tag of the transgenic proteins, and are representative of three replicates.

**Additional file 2 Figure S2.** Abi levels perturb midline crossing over phenotypes in *Abl* mutants. Shown here are late-stage embryonic nerve cords stained with mAb 1D4. (A) Wild-type embryo. (B) *Abl* homozygotes have occasional midline crossing overs. These are rescued by expression of (C) wild-type *Abl* with *1407-Gal4*, but not (D) *AblΔ1Q.* (E-F) Midline crossing overs are increased with *AblΔ1Q* with heterozygous loss of *Abi.* (G-H) Overexpression of *Abi* increases midline crossing overs, and these remain high when *AblΔ1Q* is also expressed.

**Additional file 3 Table S1.** Mass spectrometry results from GST-1Q pulldown.

**Additional file 4 Table S2**. ISNb stop short counts in *Abl* mutants expressing *Abl* transgenes, with perturbation of WRC-related genes**.** Transgenes are expressed with *1407-Gal4* in conjunction with heterozygous loss of *Hem,* gain of *Sra-1,* loss or gain of *Abi,* or gain of *trio* (*UAS-trio.B*).

**Additional file 5 Table S3**. ISNb stop short counts in *Abl* mutants expressing *Abl* transgenes, with heterozygous loss of *Sra-1* and *trio*, or gain of *trio.* Transgenes are expressed with *1407-Gal4* in conjunction with heterozygous loss of *Hem* or *Sra-1.* For *Abl*^*4/4*^ embryos, transgenes are expressed with *elav-Gal4* with gain of *trio.* *The *UAS-trio* (*UAS-trio122*) transgene used here is previously published in Kannan et al., 2017. Note that the direction of the crosses in this table is reversed compared to **Table S2** due to a balancer-induced maternal effect (see materials & methods).

**Additional file 6 Table S4.** Midline crossing over counts in *Abl* mutants expressing *Abl* transgenes, with perturbation of WRC-related genes. Transgenes are expressed with *1407-Gal4* in conjunction with heterozygous loss of *Hem,* gain of *Sra-1,* loss or gain of *Abi,* or gain of *trio* (*UAS-trio.B*).

**Additional file 7 Table S5**. Midline crossing over counts in *Abl* mutants expressing *Abl* transgenes, with perturbation of WRC-related genes. Transgenes are expressed with *1407-Gal4* in conjunction with heterozygous loss of *Hem* or *Sra-1.* Note that the direction of the crosses in this table is reversed compared to Table S2 due to a balancer-induced maternal effect (see methods).

**Additional file 8 Table S6**. Midline crossing over counts in *Abl* mutants expressing *Abl* transgenes with *elav-Gal4*, with heterozygous loss and overexpression of *ena*.

## Data Availability

Mass spectrometry data for GST-1Q pulldowns are available in the Mendeley Data repository, at https://data.mendeley.com/datasets/mw478mgmzs/1 (DOI: 10.17632/mw478mgmzs.1). Other data generated or analysed during this study are included in this published article and its supplementary information files.

## Abbreviations

Abl: Abelson tyrosine kinase
WRC: WAVE regulatory complex
Sra-1: Specifically Rac1-associated protein 1
Abi: Abelson-interacting protein
ena: Enabled
GEF: Guanine exchange factor
CTD: C-terminal domain
1Q: First quarter
1E: First eighth
2E: Second eighth
P: PxxP motif
ISNb: Intersegmental nerve b
SNa: Segmental nerve a
