# Supplementary figures and images for: The first quarter of the C-terminal domain of Abelson regulates the WAVE regulatory complex and Enabled in axon guidance
Source: Neural Dev. 2020 May 2;15:7. doi: 10.1186/s13064-020-00144-8 (PMC7196227; doi:10.1186/s13064-020-00144-8)

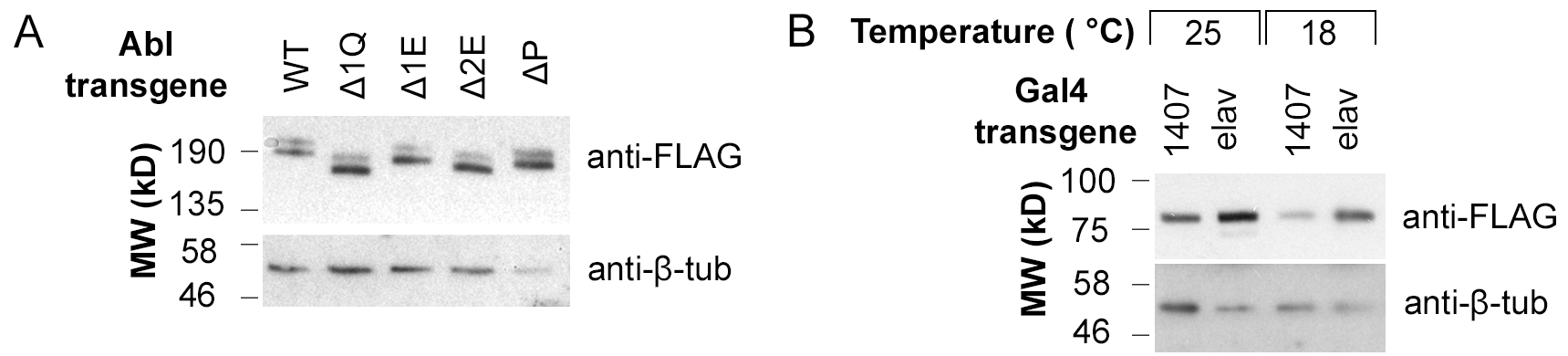

Supplement: Supplementary file 1 — Additional file 1 Figure S1. Expression levels of Abl transgenes and Gal4 drivers. (A) Expression levels of Abl transgenes, as assessed by western blots from 3rd instar CNS expressing transgenes with 1407-Gal4. (B) Comparison of expression levels of 1407-Gal4 and elav-Gal4 at 2 temperatures. The drivers were used to drive expression of UAS-RFP in the CNS of 3rd instar larvae. All immunoblotting was carried out against the C-terminal FLAG tag of the transgenic proteins, and are representative of three replicates. [file 13064_2020_144_MOESM1_ESM.tif]

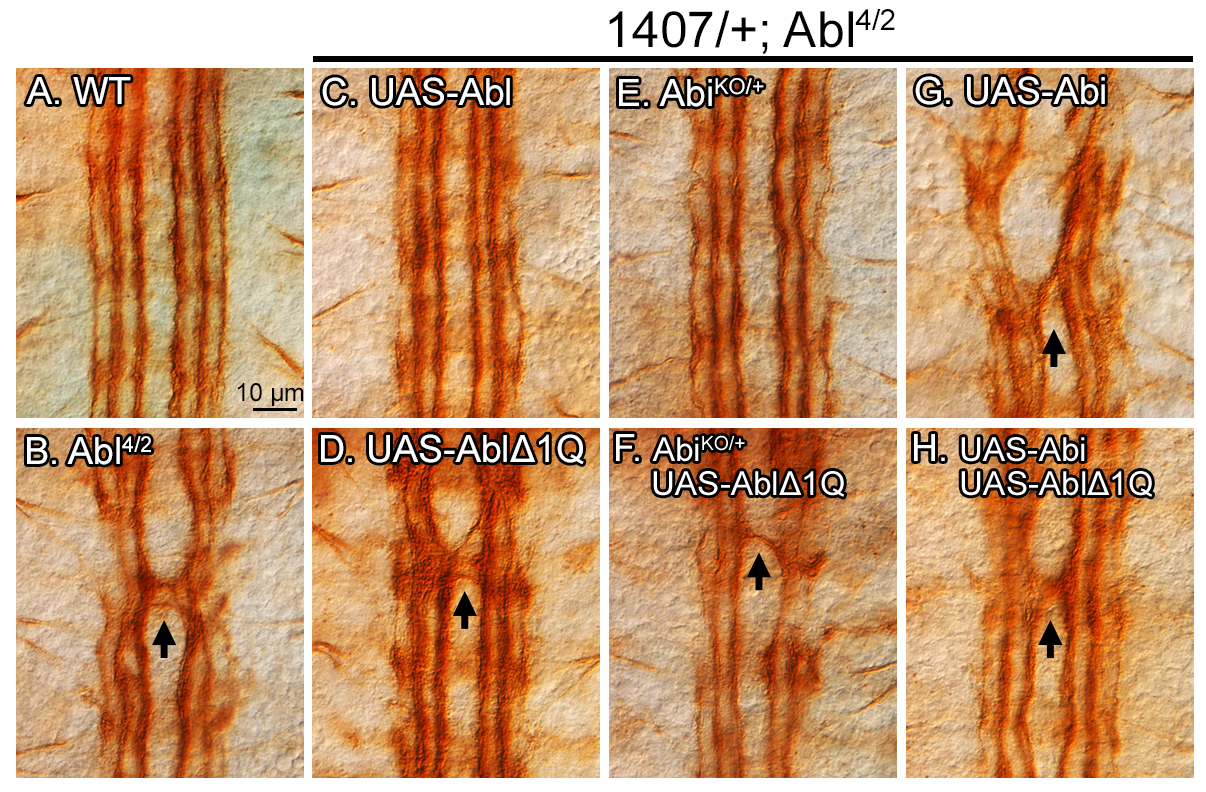

Supplement: Supplementary file 2 — Additional file 2 Figure S2. Abi levels perturb midline crossing over phenotypes in Abl mutants. Shown here are late-stage embryonic nerve cords stained with mAb 1D4. (A) Wild-type embryo. (B) Abl homozygotes have occasional midline crossing overs. These are rescued by expression of (C) wild-type Abl with 1407-Gal4, but not (D) AblΔ1Q. (E-F) Midline crossing overs are increased with AblΔ1Q with heterozygous loss of Abi. (G-H) Overexpression of Abi increases midline crossing overs, and these remain high when AblΔ1Q is also expressed. [file 13064_2020_144_MOESM2_ESM.tif]
