# Supplementary material for: The first quarter of the C-terminal domain of Abelson regulates the WAVE regulatory complex and Enabled in axon guidance
Source: Neural Dev. 2020 May 2;15:7. doi: 10.1186/s13064-020-00144-8 (PMC7196227; doi:10.1186/s13064-020-00144-8)
Supplement: Supplementary file 3 — Additional file 3 Table S1. Mass spectrometry results from GST-1Q pulldown. [file 13064_2020_144_MOESM3_ESM.docx]

|  |  |  |  |  | **Spectral count** | | | | | |
| --- | --- | --- | --- | --- | --- | --- | --- | --- | --- | --- |
| **Protein** | **Accession Number** | **MW (kD)** | **T-Test (P-Value): *(p <= 0.00228)** | **Quan-tita-tive Pro-file** | **GST-1Q pulldown** | | | **GST pulldown** | | |
|  |  |  |  |  | **1** | **2** | **3** | **1** | **2** | **3** |
| Tyrosine-protein kinase Abl | ABL_DROME | 172 | 0.0003 | 1Q high | 28 | 24 | 29 | 6 | 5 | 8 |
| Cytoplasmic FMR1-interacting protein | CYFIP_DROME | 149 | 0.00044 | 1Q high | 19 | 26 | 25 | 0 | 0 | 0 |
| Membrane-associated protein Hem | HEM_DROME | 129 | 0.0016 | 1Q high | 19 | 29 | 29 | 1 | 0 | 0 |
| 60S ribosomal protein L7a | RL7A_DROME | 31 | 0.0019 | 1Q high | 8 | 12 | 12 | 0 | 1 | 1 |
| 60S ribosomal protein L8 | RL8_DROME | 28 | < 0.00010 | 1Q high | 7 | 8 | 7 | 0 | 0 | 0 |
| 40S ribosomal protein S4 | RS4_DROME | 29 | 0.0013 | 1Q high | 6 | 9 | 9 | 0 | 0 | 0 |
| Probable elongation factor 1-delta | EF1D_DROME | 29 | 0.00056 | 1Q high | 4 | 3 | 3 | 0 | 0 | 0 |
| ATP synthase subunit beta, mitochondrial | ATPB_DROME | 54 | < 0.00010 | 1Q low | 1 | 1 | 2 | 14 | 12 | 13 |
| ATP synthase subunit alpha, mitochondrial | ATPA_DROME | 59 | 0.0042 |  | 0 | 0 | 0 | 13 | 11 | 7 |
| 60S ribosomal protein L7 | RL7_DROME | 30 | 0.0051 |  | 8 | 14 | 9 | 0 | 0 | 0 |
| LanC-like protein 3 homolog | LANC3_DROME | 48 | 0.0053 |  | 8 | 7 | 9 | 4 | 4 | 5 |
| Glutathione S-transferase S1 | GSTS1_DROME | 28 | 0.0065 |  | 36 | 35 | 38 | 25 | 14 | 18 |
| 40S ribosomal protein S6 | RS6_DROME | 28 | 0.0065 |  | 7 | 9 | 11 | 3 | 3 | 3 |
| Actin-5C | ACT1_DROME | 42 | 0.0093 |  | 27 | 26 | 23 | 18 | 15 | 19 |
| La-related protein 1 | LARP_DROME | 178 | 0.016 |  | 2 | 3 | 2 | 1 | 1 | 1 |
| 26S proteasome non-ATPase regulatory subunit 6 | PSMD6_DROME | 45 | 0.017 |  | 7 | 10 | 9 | 5 | 3 | 5 |
| Heat shock 70 kDa protein cognate 3 | HSP7C_DROME | 72 | 0.018 |  | 43 | 51 | 57 | 20 | 12 | 30 |
| Heat shock 70 kDa protein cognate 4 | HSP7D_DROME | 71 | 0.031 |  | 76 | 111 | 106 | 40 | 29 | 68 |
| T-complex protein 1 subunit alpha | TCPA_DROME | 60 | 0.033 |  | 3 | 8 | 7 | 0 | 0 | 2 |
| RNA-binding protein lark | LARK_DROME | 40 | 0.033 |  | 2 | 3 | 5 | 0 | 1 | 0 |
| Ubiquitin carboxyl-terminal hydrolase 7 | UBP7_DROME | 130 | 0.034 |  | 5 | 6 | 4 | 2 | 0 | 3 |
| ADP,ATP carrier protein | ADT_DROME | 34 | 0.035 |  | 12 | 21 | 18 | 6 | 2 | 10 |
| Eukaryotic translation initiation factor 3 subunit H | EIF3H_DROME | 38 | 0.035 |  | 3 | 3 | 5 | 2 | 1 | 1 |
| 60S ribosomal protein L3 | RL3_DROME | 47 | 0.038 |  | 4 | 6 | 1 | 11 | 7 | 11 |
| Heterogeneous nuclear ribonucleoprotein A1 | ROA1_DROME | 39 | 0.039 |  | 1 | 3 | 4 | 0 | 0 | 0 |
| Guanine nucleotide-binding protein subunit beta-like protein | GBLP_DROME | 36 | 0.041 |  | 14 | 16 | 15 | 11 | 3 | 9 |
| Eukaryotic translation initiation factor 3 subunit M | EIF3M_DROME | 44 | 0.041 |  | 4 | 9 | 8 | 2 | 2 | 3 |
| 60S ribosomal protein L5 | RL5_DROME | 34 | 0.047 |  | 15 | 16 | 19 | 14 | 9 | 11 |
| 60S ribosomal protein L15 | RL15_DROME | 24 | 0.055 |  | 1 | 3 | 3 | 0 | 0 | 1 |
| Eukaryotic translation initiation factor 2 subunit 3 | IF2G_DROME | 51 | 0.057 |  | 0 | 0 | 0 | 4 | 2 | 1 |
| Probable aminoacyl tRNA synthase complex-interacting multifunctional protein 2 | AIMP2_DROME | 37 | 0.06 |  | 5 | 6 | 7 | 2 | 2 | 5 |
| 40S ribosomal protein S3 | RS3_DROME | 27 | 0.067 |  | 26 | 31 | 31 | 21 | 11 | 24 |
| 26S proteasome non-ATPase regulatory subunit 14 | PSDE_DROME | 34 | 0.07 |  | 4 | 6 | 5 | 3 | 2 | 4 |
| Probable glutamine--tRNA ligase | SYQ_DROME | 88 | 0.081 |  | 4 | 6 | 8 | 3 | 2 | 4 |
| Succinyl-CoA ligase [ADP/GDP-forming] subunit alpha, mitochondrial | SUCA_DROME | 34 | 0.083 |  | 1 | 7 | 7 | 1 | 0 | 0 |
| Nascent polypeptide-associated complex subunit alpha | NACA_DROME | 23 | 0.089 |  | 4 | 5 | 4 | 2 | 2 | 4 |
| 26S protease regulatory subunit 8 | PRS8_DROME | 46 | 0.091 |  | 7 | 6 | 8 | 5 | 3 | 6 |
| Ran GTPase-activating protein | RAGP1_DROME | 66 | 0.091 |  | 1 | 5 | 2 | 0 | 0 | 0 |
| GTP-binding protein 128up | 128UP_DROME | 41 | 0.1 |  | 1 | 2 | 4 | 0 | 0 | 1 |
| ATP synthase subunit gamma, mitochondrial | ATPG_DROME | 33 | 0.11 |  | 1 | 7 | 3 | 0 | 0 | 0 |
| 40S ribosomal protein S3a | RS3A_DROME | 30 | 0.12 |  | 16 | 20 | 20 | 13 | 7 | 17 |
| 14-3-3 protein epsilon | 1433E_DROME | 30 | 0.12 |  | 1 | 15 | 8 | 0 | 0 | 0 |
| Actin-related protein 1 | ACTZ_DROME | 43 | 0.12 |  | 1 | 2 | 2 | 2 | 3 | 4 |
| Glycogen [starch] synthase | GYS_DROME | 82 | 0.12 |  | 1 | 3 | 3 | 1 | 1 | 1 |
| Serine/threonine-protein phosphatase alpha-2 isoform | PP12_DROME | 35 | 0.12 |  | 0 | 5 | 4 | 0 | 0 | 0 |
| Annexin B10 | ANX10_DROME | 36 | 0.12 |  | 0 | 4 | 3 | 0 | 0 | 0 |
| 60 kDa heat shock protein, mitochondrial | CH60_DROME | 61 | 0.13 |  | 2 | 4 | 5 | 1 | 0 | 3 |
| Casein kinase II subunit alpha | CSK2A_DROME | 40 | 0.13 |  | 1 | 5 | 2 | 0 | 0 | 1 |
| Failed axon connections | FAXC_DROME | 47 | 0.13 |  | 0 | 6 | 4 | 0 | 0 | 0 |
| Protein l(2)37Cc | L2CC_DROME | 30 | 0.14 |  | 0 | 8 | 5 | 0 | 0 | 0 |
| Probable arginine--tRNA ligase, cytoplasmic | SYRC_DROME | 76 | 0.15 |  | 1 | 9 | 7 | 0 | 0 | 3 |
| Clathrin light chain | CLC_DROME | 24 | 0.15 |  | 1 | 1 | 3 | 0 | 0 | 1 |
| Inorganic pyrophosphatase | IPYR_DROME | 38 | 0.16 |  | 8 | 10 | 10 | 9 | 2 | 6 |
| Probable medium-chain specific acyl-CoA dehydrogenase, mitochondrial | ACADM_DROME | 46 | 0.16 |  | 5 | 12 | 10 | 4 | 4 | 7 |
| NADPH--cytochrome P450 reductase | NCPR_DROME | 76 | 0.16 |  | 0 | 4 | 4 | 0 | 0 | 1 |
| Protein PTCD3 homolog, mitochondrial | PTCD3_DROME | 74 | 0.16 |  | 0 | 4 | 2 | 0 | 0 | 0 |
| 60S ribosomal protein L4 | RL4_DROME | 45 | 0.17 |  | 19 | 31 | 26 | 16 | 16 | 23 |
| Elongation factor 1-gamma | EF1G_DROME | 49 | 0.17 |  | 11 | 16 | 13 | 10 | 4 | 12 |
| 60S acidic ribosomal protein P0 | RLA0_DROME | 34 | 0.18 |  | 12 | 15 | 15 | 10 | 8 | 14 |
| Eukaryotic translation initiation factor 3 subunit I | EIF3I_DROME | 36 | 0.18 |  | 1 | 7 | 4 | 1 | 0 | 2 |
| ATP-dependent 6-phosphofructokinase | PFKA_DROME | 87 | 0.18 |  | 0 | 4 | 3 | 0 | 0 | 1 |
| Calcium-binding mitochondrial carrier protein Aralar1 | CMC_DROME | 77 | 0.18 |  | 1 | 7 | 5 | 0 | 0 | 3 |
| Voltage-dependent anion-selective channel | VDAC_DROME | 31 | 0.18 |  | 0 | 5 | 2 | 0 | 0 | 0 |
| Glyceraldehyde-3-phosphate dehydrogenase 2 | G3P2_DROME | 35 | 0.19 |  | 18 | 29 | 24 | 17 | 11 | 22 |
| Eukaryotic translation initiation factor 3 subunit B | EIF3B_DROME | 80 | 0.19 |  | 3 | 11 | 9 | 2 | 2 | 6 |
| Krueppel homolog 2 | KRH2_DROME | 31 | 0.19 |  | 0 | 5 | 3 | 0 | 0 | 1 |
| Proteasome subunit alpha type-1 | PSA1_DROME | 31 | 0.2 |  | 7 | 14 | 13 | 7 | 5 | 10 |
| 26S proteasome non-ATPase regulatory subunit 7 | PSMD7_DROME | 38 | 0.21 |  | 3 | 11 | 5 | 3 | 2 | 3 |
| Heat shock protein 26 | HSP26_DROME | 23 | 0.21 |  | 0 | 3 | 1 | 0 | 0 | 0 |
| Tubulin beta-1 chain | TBB1_DROME | 50 | 0.23 |  | 6 | 6 | 6 | 18 | 7 | 8 |
| rRNA 2'-O-methyltransferase fibrillarin | FBRL_DROME | 35 | 0.23 |  | 3 | 7 | 9 | 3 | 3 | 5 |
| Polyadenylate-binding protein 2 | PABP2_DROME | 25 | 0.23 |  | 2 | 2 | 0 | 4 | 2 | 2 |
| Caprin homolog | CAPR1_DROME | 104 | 0.23 |  | 0 | 5 | 4 | 0 | 0 | 2 |
| Proteasomal ubiquitin receptor ADRM1 homolog | ADRM1_DROME | 42 | 0.23 |  | 0 | 1 | 0 | 3 | 0 | 2 |
| S-methyl-5'-thioadenosine phosphorylase | MTAP_DROME | 32 | 0.23 |  | 0 | 2 | 3 | 0 | 0 | 1 |
| Fructose-bisphosphate aldolase | ALF_DROME | 39 | 0.24 |  | 5 | 20 | 20 | 5 | 3 | 13 |
| Putative ATP-dependent RNA helicase me31b | DDX6_DROME | 52 | 0.24 |  | 0 | 0 | 1 | 4 | 0 | 2 |
| Lipid storage droplets surface-binding protein 2 | LSD2_DROME | 38 | 0.25 |  | 0 | 1 | 1 | 3 | 0 | 4 |
| Casein kinase I isoform alpha | KC1A_DROME | 40 | 0.25 |  | 1 | 1 | 3 | 1 | 0 | 1 |
| Eukaryotic translation initiation factor 3 subunit G-1 | EI3G1_DROME | 30 | 0.27 |  | 0 | 3 | 3 | 1 | 0 | 1 |
| Tropomyosin-1, isoforms 33/34 | TPM4_DROME | 55 | 0.28 |  | 1 | 4 | 4 | 1 | 0 | 3 |
| Proliferating cell nuclear antigen | PCNA_DROME | 29 | 0.28 |  | 3 | 5 | 6 | 4 | 0 | 4 |
| C-terminal-binding protein | CTBP_DROME | 51 | 0.28 |  | 2 | 3 | 6 | 1 | 2 | 3 |
| Adenosylhomocysteinase | SAHH_DROME | 47 | 0.29 |  | 0 | 2 | 0 | 3 | 0 | 4 |
| ATP-dependent RNA helicase bel | DDX3_DROME | 85 | 0.3 |  | 2 | 14 | 15 | 5 | 2 | 8 |
| Tubulin alpha-1 chain | TBA1_DROME (+1) | 50 | 0.31 |  | 1 | 6 | 7 | 11 | 5 | 7 |
| UDP-glucose:glycoprotein glucosyltransferase | UGGG_DROME | 174 | 0.32 |  | 1 | 10 | 9 | 2 | 1 | 6 |
| V-type proton ATPase catalytic subunit A isoform 2 | VATA2_DROME | 68 | 0.33 |  | 5 | 17 | 15 | 4 | 1 | 14 |
| Eukaryotic translation initiation factor 2 subunit 2 | IF2B_DROME | 35 | 0.33 |  | 1 | 5 | 6 | 1 | 1 | 4 |
| Probable phosphoserine aminotransferase | SERC_DROME | 40 | 0.33 |  | 1 | 6 | 5 | 1 | 1 | 4 |
| Ribosome production factor 2 homolog | RPF2_DROME | 36 | 0.33 |  | 1 | 1 | 4 | 0 | 0 | 2 |
| Protein Mo25 | MO25_DROME | 39 | 0.33 |  | 0 | 3 | 3 | 0 | 0 | 2 |
| Signal recognition particle subunit SRP68 | SRP68_DROME | 69 | 0.33 |  | 0 | 3 | 3 | 0 | 0 | 2 |
| DNA replication licensing factor Mcm3 | MCM3_DROME | 91 | 0.35 |  | 1 | 7 | 6 | 3 | 0 | 4 |
| Fragile X mental retardation syndrome-related protein 1 | FMR1_DROME | 76 | 0.35 |  | 1 | 4 | 3 | 0 | 1 | 3 |
| Regulator of chromosome condensation | RCC1_DROME | 59 | 0.35 |  | 0 | 1 | 3 | 0 | 0 | 1 |
| Putative oxidoreductase GLYR1 homolog | GLYR1_DROME | 65 | 0.35 |  | 0 | 3 | 2 | 1 | 0 | 1 |
| Protein SET | SET_DROME | 31 | 0.35 |  | 0 | 3 | 1 | 0 | 0 | 1 |
| Zinc finger protein on ecdysone puffs | PEP_DROME | 78 | 0.37 |  | 2 | 6 | 6 | 4 | 1 | 4 |
| Phenylalanine--tRNA ligase beta subunit | SYFB_DROME | 66 | 0.37 |  | 1 | 10 | 5 | 0 | 0 | 6 |
| Selenide, water dikinase | SPS1_DROME | 43 | 0.37 |  | 1 | 5 | 5 | 1 | 1 | 4 |
| Pyruvate kinase | KPYK_DROME | 57 | 0.37 |  | 0 | 0 | 0 | 3 | 0 | 0 |
| Exportin-2 | XPO2_DROME | 110 | 0.39 |  | 1 | 14 | 9 | 3 | 0 | 8 |
| Vitellogenin-3 | VIT3_DROME | 46 | 0.4 |  | 22 | 40 | 38 | 26 | 18 | 35 |
| Enolase | ENO_DROME | 54 | 0.41 |  | 3 | 8 | 3 | 7 | 5 | 7 |
| Replication protein A 70 kDa DNA-binding subunit | RFA1_DROME | 67 | 0.41 |  | 1 | 6 | 5 | 0 | 1 | 5 |
| Ubiquitin-40S ribosomal protein S27a | RS27A_DROME | 18 | 0.42 |  | 1 | 3 | 4 | 3 | 3 | 5 |
| Probable transaldolase | TALDO_DROME | 37 | 0.44 |  | 0 | 3 | 3 | 1 | 0 | 2 |
| Myosin heavy chain, muscle | MYSA_DROME | 224 | 0.46 |  | 0 | 2 | 6 | 0 | 0 | 3 |
| Probable citrate synthase, mitochondrial | CISY_DROME | 52 | 0.46 |  | 0 | 5 | 5 | 1 | 0 | 4 |
| Glutamine synthetase 1, mitochondrial | GLNA1_DROME | 44 | 0.46 |  | 0 | 4 | 5 | 0 | 0 | 4 |
| Eukaryotic translation initiation factor 3 subunit E | EIF3E_DROME | 51 | 0.47 |  | 6 | 8 | 8 | 8 | 3 | 7 |
| Succinate dehydrogenase [ubiquinone] flavoprotein subunit, mitochondrial | SDHA_DROME | 72 | 0.48 |  | 1 | 7 | 6 | 0 | 0 | 7 |
| Apolipophorins | APLP_DROME | 373 | 0.49 |  | 5 | 32 | 34 | 13 | 5 | 27 |
| Probable isocitrate dehydrogenase [NAD] subunit alpha, mitochondrial | IDH3A_DROME | 41 | 0.49 |  | 2 | 7 | 11 | 5 | 4 | 5 |
| DNA-binding protein modulo | MODU_DROME | 60 | 0.5 |  | 5 | 13 | 12 | 6 | 2 | 13 |
| ATP-dependent RNA helicase WM6 | DX39B_DROME | 49 | 0.51 |  | 1 | 6 | 4 | 5 | 3 | 7 |
| T-complex protein 1 subunit gamma | TCPG_DROME | 59 | 0.51 |  | 0 | 4 | 0 | 0 | 0 | 1 |
| Cysteine--tRNA ligase, cytoplasmic | SYCC_DROME | 84 | 0.52 |  | 0 | 3 | 3 | 0 | 0 | 3 |
| Heat shock 70 kDa protein cognate 5 | HSP7E_DROME | 74 | 0.53 |  | 1 | 12 | 8 | 3 | 0 | 8 |
| Glycerol-3-phosphate dehydrogenase [NAD(+)], cytoplasmic | GPDA_DROME | 40 | 0.53 |  | 0 | 5 | 4 | 1 | 0 | 4 |
| 26S proteasome non-ATPase regulatory subunit 11 | PSD11_DROME | 47 | 0.54 |  | 9 | 7 | 10 | 7 | 6 | 10 |
| V-type proton ATPase subunit C | VATC_DROME | 92 | 0.54 |  | 1 | 3 | 3 | 0 | 0 | 4 |
| Nucleoplasmin-like protein | NLP_DROME | 17 | 0.54 |  | 0 | 4 | 3 | 1 | 0 | 3 |
| Eukaryotic translation initiation factor 3 subunit A | EIF3A_DROME | 134 | 0.55 |  | 7 | 23 | 23 | 14 | 8 | 19 |
| Elongation factor 1-alpha 1 | EF1A1_DROME | 50 | 0.55 |  | 12 | 19 | 16 | 17 | 17 | 17 |
| Protein rigor mortis | RIG_DROME | 138 | 0.55 |  | 0 | 4 | 2 | 0 | 0 | 3 |
| COP9 signalosome complex subunit 4 | CSN4_DROME | 46 | 0.55 |  | 0 | 2 | 3 | 0 | 0 | 2 |
| Glycogen phosphorylase | PYG_DROME | 97 | 0.56 |  | 5 | 21 | 16 | 8 | 2 | 19 |
| Neurotactin | NRT_DROME | 93 | 0.56 |  | 0 | 3 | 2 | 1 | 0 | 2 |
| Lamin Dm0 | LAM0_DROME | 71 | 0.57 |  | 1 | 3 | 5 | 2 | 0 | 4 |
| Phenoloxidase 2 | PPO2_DROME | 79 | 0.58 |  | 0 | 3 | 1 | 0 | 0 | 2 |
| Vitellogenin-1 | VIT1_DROME | 49 | 0.6 |  | 18 | 29 | 23 | 29 | 15 | 38 |
| Eukaryotic translation initiation factor 3 subunit C | EIF3C_DROME | 106 | 0.6 |  | 2 | 16 | 15 | 8 | 1 | 14 |
| Protein amalgam | AMAL_DROME | 36 | 0.6 |  | 0 | 5 | 6 | 2 | 0 | 5 |
| 60S ribosomal protein L22 | RL22_DROME | 31 | 0.61 |  | 11 | 15 | 14 | 13 | 8 | 15 |
| DNA-directed RNA polymerase II subunit RPB2 | RPB2_DROME | 134 | 0.61 |  | 0 | 2 | 2 | 3 | 0 | 3 |
| Neurochondrin homolog | NCDN_DROME | 82 | 0.61 |  | 0 | 3 | 4 | 0 | 0 | 4 |
| Clathrin heavy chain | CLH_DROME | 191 | 0.62 |  | 14 | 28 | 31 | 17 | 9 | 33 |
| Protein krasavietz | PKRA_DROME | 49 | 0.64 |  | 2 | 6 | 2 | 4 | 2 | 7 |
| DNA replication licensing factor MCM4 | MCM4_DROME | 97 | 0.64 |  | 0 | 1 | 2 | 1 | 1 | 3 |
| Eukaryotic translation initiation factor 3 subunit D-1 | EI3D1_DROME | 64 | 0.64 |  | 0 | 3 | 3 | 0 | 1 | 3 |
| Cullin-associated NEDD8-dissociated protein 1 | CAND1_DROME | 139 | 0.65 |  | 1 | 7 | 6 | 3 | 0 | 7 |
| Dynamin | DYN_DROME | 98 | 0.67 |  | 0 | 5 | 4 | 1 | 0 | 5 |
| Elongation factor 2 | EF2_DROME | 94 | 0.68 |  | 20 | 42 | 38 | 22 | 15 | 47 |
| 39 kDa FK506-binding nuclear protein | FKB39_DROME | 39 | 0.68 |  | 0 | 3 | 4 | 2 | 0 | 3 |
| 40S ribosomal protein SA | RSSA_DROME | 35 | 0.69 |  | 9 | 10 | 10 | 11 | 6 | 10 |
| Alpha-actinin, sarcomeric | ACTN_DROME | 107 | 0.69 |  | 0 | 7 | 7 | 3 | 0 | 7 |
| Eukaryotic initiation factor 4A | IF4A_DROME | 46 | 0.72 |  | 8 | 23 | 17 | 14 | 9 | 19 |
| Alanine--tRNA ligase, cytoplasmic | SYAC_DROME | 108 | 0.72 |  | 0 | 10 | 10 | 5 | 0 | 10 |
| Tripeptidyl-peptidase 2 | TPP2_DROME | 159 | 0.74 |  | 1 | 22 | 18 | 7 | 7 | 19 |
| DNA replication licensing factor Mcm7 | MCM7_DROME | 81 | 0.74 |  | 1 | 6 | 3 | 2 | 1 | 5 |
| Oxygen-dependent coproporphyrinogen-III oxidase | HEM6_DROME | 44 | 0.74 |  | 0 | 1 | 3 | 2 | 1 | 2 |
| Calcium-transporting ATPase sarcoplasmic/endoplasmic reticulum type | ATC1_DROME | 112 | 0.75 |  | 1 | 14 | 7 | 6 | 0 | 11 |
| Actin-87E | ACT5_DROME | 42 | 0.76 |  | 0 | 3 | 1 | 0 | 0 | 4 |
| Protein hu-li tai shao | HTS_DROME | 128 | 0.77 |  | 0 | 3 | 1 | 1 | 0 | 2 |
| Coatomer subunit beta' | COPB2_DROME | 103 | 0.78 |  | 1 | 4 | 3 | 3 | 1 | 3 |
| Neuroglian | NRG_DROME | 144 | 0.78 |  | 0 | 2 | 2 | 2 | 0 | 3 |
| Mitochondrial import receptor subunit TOM40 homolog 1 | TO401_DROME | 36 | 0.78 |  | 0 | 2 | 2 | 2 | 0 | 3 |
| Transitional endoplasmic reticulum ATPase TER94 | TERA_DROME | 89 | 0.8 |  | 8 | 34 | 25 | 18 | 10 | 31 |
| DNA damage-binding protein 1 | DDB1_DROME | 126 | 0.8 |  | 3 | 9 | 8 | 6 | 3 | 14 |
| Multifunctional protein ADE2 | PUR6_DROME | 47 | 0.8 |  | 0 | 2 | 3 | 0 | 1 | 3 |
| Obg-like ATPase 1 | OLA1_DROME | 45 | 0.8 |  | 1 | 2 | 2 | 2 | 0 | 4 |
| Vesicle-fusing ATPase 2 | NSF2_DROME | 83 | 0.8 |  | 0 | 2 | 2 | 0 | 0 | 3 |
| Ribonucleoside-diphosphate reductase large subunit | RIR1_DROME | 92 | 0.81 |  | 2 | 8 | 8 | 6 | 2 | 8 |
| DNA replication licensing factor Mcm5 | MCM5_DROME | 82 | 0.81 |  | 1 | 6 | 9 | 6 | 4 | 8 |
| Arginine kinase | KARG_DROME | 40 | 0.81 |  | 0 | 8 | 5 | 2 | 3 | 6 |
| Paramyosin, long form | MYSP1_DROME | 102 | 0.83 |  | 1 | 9 | 11 | 4 | 2 | 12 |
| Sodium/potassium-transporting ATPase subunit alpha | ATNA_DROME | 116 | 0.83 |  | 0 | 9 | 8 | 3 | 0 | 11 |
| Importin subunit beta | IMB_DROME | 99 | 0.83 |  | 2 | 9 | 7 | 5 | 2 | 9 |
| MICOS complex subunit Mic60 | MIC60_DROME | 82 | 0.83 |  | 0 | 1 | 2 | 0 | 0 | 4 |
| Bifunctional glutamate/proline--tRNA ligase | SYEP_DROME | 189 | 0.84 |  | 2 | 10 | 10 | 8 | 2 | 15 |
| DNA topoisomerase 2 | TOP2_DROME | 164 | 0.84 |  | 1 | 7 | 5 | 3 | 2 | 10 |
| Dipeptidyl peptidase 3 | DPP3_DROME | 89 | 0.85 |  | 1 | 13 | 7 | 3 | 2 | 13 |
| NADH-ubiquinone oxidoreductase 75 kDa subunit, mitochondrial | NDUS1_DROME | 79 | 0.85 |  | 1 | 8 | 8 | 5 | 1 | 9 |
| Presequence protease, mitochondrial | PREP_DROME | 119 | 0.85 |  | 1 | 3 | 3 | 3 | 0 | 5 |
| DNA replication licensing factor Mcm6 | MCM6_DROME | 92 | 0.85 |  | 1 | 3 | 4 | 2 | 0 | 5 |
| Kinesin heavy chain | KINH_DROME | 110 | 0.86 |  | 0 | 9 | 9 | 6 | 2 | 8 |
| Alcohol dehydrogenase class-3 | ADHX_DROME | 40 | 0.86 |  | 0 | 11 | 5 | 6 | 2 | 6 |
| 26S proteasome non-ATPase regulatory subunit 1 | PSMD1_DROME | 113 | 0.89 |  | 9 | 25 | 21 | 16 | 10 | 26 |
| Chromatin-remodeling complex ATPase chain Iswi | ISWI_DROME | 119 | 0.89 |  | 0 | 5 | 5 | 4 | 0 | 5 |
| Coatomer subunit gamma | COPG_DROME | 98 | 0.89 |  | 3 | 4 | 6 | 3 | 1 | 8 |
| Coatomer subunit beta | COPB_DROME | 107 | 0.89 |  | 1 | 6 | 5 | 3 | 1 | 7 |
| Exportin-1 | XPO1_DROME | 123 | 0.9 |  | 0 | 12 | 10 | 7 | 1 | 12 |
| Protein clueless | CLU_DROME | 161 | 0.9 |  | 0 | 4 | 3 | 1 | 0 | 7 |
| Eukaryotic translation initiation factor 2 subunit 1 | IF2A_DROME | 39 | 0.93 |  | 1 | 9 | 9 | 9 | 2 | 9 |
| Polyadenylate-binding protein | PABP_DROME | 70 | 0.94 |  | 4 | 14 | 15 | 10 | 7 | 15 |
| Phosphoglycerate kinase | PGK_DROME | 44 | 0.94 |  | 1 | 12 | 12 | 10 | 5 | 11 |
| Moesin/ezrin/radixin homolog 1 | MOEH_DROME | 68 | 0.95 |  | 1 | 7 | 9 | 2 | 1 | 15 |
| Heat shock protein 83 | HSP83_DROME | 82 | 0.98 |  | 16 | 48 | 45 | 43 | 15 | 50 |
| Vitellogenin-2 | VIT2_DROME | 50 | 1 |  | 19 | 41 | 32 | 28 | 22 | 42 |
| AP-2 complex subunit alpha | AP2A_DROME | 106 | 1 |  | 0 | 2 | 1 | 0 | 0 | 3 |
| Phosphoribosylformylglycin-amidine synthase | PUR4_DROME | 148 | 1 |  | 0 | 2 | 4 | 2 | 0 | 4 |
| Fasciclin-1 | FAS1_DROME | 73 | 1 |  | 0 | 3 | 3 | 1 | 0 | 5 |
| 60S ribosomal protein L13 | RL13_DROME | 25 | 1 |  | 3 | 3 | 3 | 0 | 0 | 0 |
| Protein NASP homolog | NASP_DROME | 52 | 1 |  | 0 | 3 | 0 | 0 | 0 | 3 |
