# Supplementary material for: The first quarter of the C-terminal domain of Abelson regulates the WAVE regulatory complex and Enabled in axon guidance
Source: Neural Dev. 2020 May 2;15:7. doi: 10.1186/s13064-020-00144-8 (PMC7196227; doi:10.1186/s13064-020-00144-8)
Supplement: Supplementary file 4 — Additional file 4 Table S2. ISNb stop short counts in Abl mutants expressing Abl transgenes, with perturbation of WRC-related genes. Transgenes are expressed with 1407-Gal4 in conjunction with heterozygous loss of Hem, gain of Sra-1, loss or gain of Abi, or gain of trio (UAS-trio.B). [file 13064_2020_144_MOESM4_ESM.docx]

| **Genotype** | | | **n** | **% Hemisegments with  stop shorts** | | | **p (to matched transgene in control)** | **p (to Abl^4/2^ within group)** |
| --- | --- | --- | --- | --- | --- | --- | --- | --- |
| **Abl alleles** | **Other** | **Abl transgene** |  | **6/13** | **7/6** | **Total** |  |  |
| Abl^4/+^ |  |  | 1298 | 3.5 | 0.7 | 4.2 | - | <.0001 |
| Abl^4/2^ | controls | ∅ | 1560 | 17.5 | 1.6 | 19.1 | - | - |
|  |  | WT | 1074 | 3.8 | 0.2 | 4.0 | - | <.0001 |
|  |  | Δ1Q | 1185 | 8.5 | 0.5 | 9.0 | - | <.0001 |
|  |  | Δ1E | 361 | 4.7 | 0.3 | 5.0 | - | <.0001 |
|  |  | Δ2E | 1273 | 7.8 | 0.9 | 8.6 | - | <.0001 |
|  |  | ΔP | 1183 | 8.8 | 1.0 | 9.8 | - | <.0001 |
|  | Hem^J4-48/+^ | ∅ | 635 | 14.3 | 0.9 | 15.3 | 0.9669 | - |
|  |  | WT | 230 | 0.9 | 0.0 | 0.9 | 0.9669 | 0.0012 |
|  |  | Δ1Q | 220 | 4.5 | 0.5 | 5.0 | 1 | 0.0078 |
|  |  | Δ1E | 177 | 7.3 | 0.6 | 7.9 | 1 | 0.451 |
|  |  | Δ2E | 219 | 6.4 | 0.5 | 6.8 | 1 | 0.0778 |
|  |  | ΔP | 218 | 5.5 | 0.5 | 6.0 | 1 | 0.0272 |
|  | UAS-Sra-1 | ∅ | 236 | 10.2 | 0.8 | 11.0 | 0.1086 | - |
|  |  | WT | 181 | 6.1 | 0.0 | 6.1 | 1 | 1 |
|  |  | Δ1Q | 213 | 7.0 | 1.4 | 8.5 | 1 | 1 |
|  |  | Δ1E | 115 | 6.1 | 0.9 | 7.0 | 1 | 1 |
|  |  | Δ2E | 222 | 5.4 | 0.0 | 5.4 | 1 | 0.9669 |
|  |  | ΔP | 256 | 7.4 | 0.4 | 7.8 | 1 | 1 |
|  | Abi^KO/+^ | ∅ | 276 | 10.5 | 1.4 | 12.0 | 0.186 | - |
|  |  | WT | 167 | 3.0 | 0.0 | 3.0 | 1 | 0.091 |
|  |  | Δ1Q | 211 | 12.8 | 0.0 | 12.8 | 1 | 1 |
|  |  | Δ1E | 126 | 4.8 | 0.0 | 4.8 | 1 | 0.8793 |
|  |  | Δ2E | 298 | 7.4 | 0.0 | 7.4 | 1 | 1 |
|  |  | ΔP | 272 | 2.6 | 0.0 | 2.6 | 0.0134 | 0.0047 |
|  | UAS-Abi | ∅ | 543 | 16.8 | 1.7 | 18.4 | 1 | - |
|  |  | WT | 202 | 3.5 | 0.0 | 3.5 | 1 | 0.0002 |
|  |  | Δ1Q | 231 | 16.5 | 2.6 | 19.0 | 0.0003 | 1 |
|  |  | Δ1E | 205 | 2.9 | 0.0 | 2.9 | 1 | 0.0001 |
|  |  | Δ2E | 169 | 10.1 | 1.2 | 11.2 | 1 | 0.9669 |
|  |  | ΔP | 184 | 12.0 | 2.2 | 14.1 | 1 | 1 |
|  | UAS-trio | ∅ | 228 | 54.4 | 9.2 | 63.6 | <.0001 | - |
|  |  | WT | 314 | 5.7 | 0.0 | 5.7 | 1 | <.0001 |
|  |  | Δ1Q | 268 | 45.9 | 6.0 | 51.9 | <.0001 | 0.254 |
|  |  | Δ1E | 227 | 10.6 | 1.3 | 11.9 | 0.1005 | <.0001 |
|  |  | Δ2E | 256 | 29.7 | 3.1 | 32.8 | <.0001 | <.0001 |
|  |  | ΔP | 279 | 28.7 | 6.1 | 34.8 | <.0001 | <.0001 |
