# Supplementary material for: The first quarter of the C-terminal domain of Abelson regulates the WAVE regulatory complex and Enabled in axon guidance
Source: Neural Dev. 2020 May 2;15:7. doi: 10.1186/s13064-020-00144-8 (PMC7196227; doi:10.1186/s13064-020-00144-8)
Supplement: Supplementary file 5 — Additional file 5 Table S3. ISNb stop short counts in Abl mutants expressing Abl transgenes, with heterozygous loss of Sra-1 and trio, or gain of trio. Transgenes are expressed with 1407-Gal4 in conjunction with heterozygous loss of Hem or Sra-1. For Abl4/4 embryos, transgenes are expressed with elav-Gal4 with gain of trio. *The UAS-trio (UAS-trio122) transgene used here is previously published in Kannan et al., 2017. Note that the direction of the crosses in this table is reversed compared to Table S2 due to a balancer-induced maternal effect (see materials & methods). [file 13064_2020_144_MOESM5_ESM.docx]

| **Genotype** | | | **n** | **% Hemisegments with stop shorts** | | | **p (to matched transgene in control)** | **p (to Abl^4/2^ within group)** |
| --- | --- | --- | --- | --- | --- | --- | --- | --- |
| **Abl alleles** | **Other** | **Abl transgene** |  | **6/13** | **7/6** | **Total** |  |  |
| Abl^4/+^ |  |  | 601 | 2.2 | 0.2 | 2.3 | - | <.0001 |
| Abl^4/2^ | controls | ∅ | 552 | 16.8 | 1.3 | 18.1 | - | - |
|  |  | WT | 445 | 6.5 | 0.2 | 6.7 | - | <.0001 |
|  |  | Δ1Q | 462 | 18.6 | 1.5 | 20.1 | - | 1 |
|  |  | Δ1E | 150 | 8.0 | 0.0 | 8.0 | - | 0.0304 |
|  |  | Δ2E | 282 | 12.4 | 0.7 | 13.1 | - | 0.4554 |
|  |  | ΔP | 261 | 8.4 | 0.8 | 9.2 | - | 0.0122 |
|  | trio^1/+^ | ∅ | 600 | 18.8 | 1.5 | 20.3 | 1 | - |
|  |  | WT | 285 | 3.2 | 0.4 | 3.5 | 0.4554 | <.0001 |
|  |  | Δ1Q | 252 | 31.3 | 2.0 | 33.3 | 0.0016 | 0.0011 |
|  |  | Δ1E | 245 | 8.6 | 0.4 | 9.0 | 1 | 0.0013 |
|  |  | Δ2E | 310 | 19.0 | 2.6 | 21.6 | 0.0469 | 1 |
|  |  | ΔP | 288 | 10.1 | 0.7 | 10.8 | 1 | 0.0055 |
|  | Sra-1^Df/+^ | ∅ | 247 | 2.8 | 0.4 | 3.2 | <.0001 | - |
|  |  | WT | 254 | 2.4 | 0.0 | 2.4 | 0.0481 | 0.8246 |
|  |  | Δ1Q | 165 | 9.1 | 1.8 | 10.9 | 0.0399 | 0.0142 |
| Abl^4/4^ | control | ∅ | 244 | 17.6 | 1.2 | 18.9 | - | - |
|  | UAS-trio* | ∅ | 230 | 48.3 | 5.7 | 53.9 | - | - |
