# Supplementary material for: The first quarter of the C-terminal domain of Abelson regulates the WAVE regulatory complex and Enabled in axon guidance
Source: Neural Dev. 2020 May 2;15:7. doi: 10.1186/s13064-020-00144-8 (PMC7196227; doi:10.1186/s13064-020-00144-8)
Supplement: Supplementary file 6 — Additional file 6 Table S4. Midline crossing over counts in Abl mutants expressing Abl transgenes, with perturbation of WRC-related genes. Transgenes are expressed with 1407-Gal4 in conjunction with heterozygous loss of Hem, gain of Sra-1, loss or gain of Abi, or gain of trio (UAS-trio.B). [file 13064_2020_144_MOESM6_ESM.docx]

| **Genotype** | | | **n** | **% embryos with crossing over** | **Average crossing overs** | **p (to matched transgene in control)** | **p (to Abl^4/2^ within group)** |
| --- | --- | --- | --- | --- | --- | --- | --- |
| **Abl alleles** | **Other** | **Abl transgene** |  |  |  |  |  |
| Abl^4/+^ |  |  | 454 | 1.8 | 0.02 | - | - |
| Abl^4/2^ | ∅ | ∅ | 391 | 50.1 | 0.83 | - | <.0001 |
|  |  | WT | 435 | 7.6 | 0.11 | - | 1 |
|  |  | Δ1Q | 416 | 54.3 | 0.98 | - | <.0001 |
|  |  | Δ1E | 208 | 15.9 | 0.22 | - | <.0001 |
|  |  | Δ2E | 439 | 23.7 | 0.37 | - | <.0001 |
|  |  | ΔP | 429 | 25.4 | 0.34 | - | <.0001 |
|  | Hem^J4-48/+^ | ∅ | 86 | 39.5 | 0.85 | 1 | - |
|  |  | WT | 84 | 4.8 | 0.06 | 1 | <.0001 |
|  |  | Δ1Q | 83 | 55.4 | 1.05 | 1 | 1 |
|  |  | Δ1E | 107 | 36.4 | 0.79 | <.0001 | 1 |
|  |  | Δ2E | 85 | 49.4 | 0.76 | 0.0009 | 1 |
|  |  | ΔP | 86 | 30.2 | 0.35 | 1 | 0.0121 |
|  | UAS-Sra-1 | ∅ | 71 | 40.8 | 0.65 | 1 | - |
|  |  | WT | 73 | 13.7 | 0.19 | 1 | 0.0099 |
|  |  | Δ1Q | 76 | 39.5 | 0.51 | 0.0275 | 1 |
|  |  | Δ1E | 70 | 15.7 | 0.19 | 1 | 0.0099 |
|  |  | Δ2E | 85 | 18.8 | 0.29 | 1 | 0.1447 |
|  |  | ΔP | 77 | 23.4 | 0.39 | 1 | 1 |
|  | Abi^KO/+^ | ∅ | 99 | 28.3 | 0.55 | 0.46 | - |
|  |  | WT | 100 | 10.0 | 0.20 | 0.8582 | 0.0146 |
|  |  | Δ1Q | 86 | 72.1 | 1.52 | 0.0372 | <.0001 |
|  |  | Δ1E | 85 | 57.6 | 0.86 | <.0001 | 0.9451 |
|  |  | Δ2E | 88 | 8.0 | 0.08 | 0.0052 | 0.0002 |
|  |  | ΔP | 81 | 13.6 | 0.15 | 0.3029 | 0.0053 |
|  | UAS-Abi | ∅ | 175 | 65.7 | 1.41 | <.0001 | - |
|  |  | WT | 78 | 14.1 | 0.18 | 1 | <.0001 |
|  |  | Δ1Q | 73 | 46.6 | 1.05 | 1 | 1 |
|  |  | Δ1E | 103 | 42.7 | 0.67 | <.0001 | 0.0003 |
|  |  | Δ2E | 58 | 44.8 | 0.67 | 0.1194 | 0.0132 |
|  |  | ΔP | 62 | 41.9 | 0.69 | 0.013 | 0.014 |
|  | UAS-trio | ∅ | 81 | 97.5 | 4.59 | <.0001 | - |
|  |  | WT | 98 | 31.6 | 0.46 | <.0001 | <.0001 |
|  |  | Δ1Q | 74 | 100.0 | 4.78 | <.0001 | 1 |
|  |  | Δ1E | 87 | 74.7 | 1.86 | <.0001 | <.0001 |
|  |  | Δ2E | 88 | 70.5 | 1.78 | <.0001 | <.0001 |
|  |  | ΔP | 87 | 73.6 | 1.59 | <.0001 | <.0001 |
