# Supplementary material for: The first quarter of the C-terminal domain of Abelson regulates the WAVE regulatory complex and Enabled in axon guidance
Source: Neural Dev. 2020 May 2;15:7. doi: 10.1186/s13064-020-00144-8 (PMC7196227; doi:10.1186/s13064-020-00144-8)
Supplement: Supplementary file 7 — Additional file 7 Table S5. Midline crossing over counts in Abl mutants expressing Abl transgenes, with perturbation of WRC-related genes. Transgenes are expressed with 1407-Gal4 in conjunction with heterozygous loss of Hem or Sra-1. Note that the direction of the crosses in this table is reversed compared to Table S2 due to a balancer-induced maternal effect (see methods). [file 13064_2020_144_MOESM7_ESM.docx]

| **Genotype** | | | **n** | **% embryos with crossing over** | **Average crossing overs** | **p (to matched transgene in control)** | **p (to Abl^4/2^ within group)** |
| --- | --- | --- | --- | --- | --- | --- | --- |
| **Abl alleles** | **Other** | **Abl transgene** |  |  |  |  |  |
| Abl^4/+^ |  |  | 196 | 2.6 | 0.03 | - | <.0001 |
| Abl^4/2^ | ∅ | ∅ | 158 | 63.9 | 1.28 | - | - |
|  |  | WT | 163 | 14.7 | 0.21 | - | <.0001 |
|  |  | Δ1Q | 151 | 78.8 | 2.15 | - | 0.0005 |
|  |  | Δ1E | 113 | 19.5 | 0.23 | - | <.0001 |
|  |  | Δ2E | 90 | 78.9 | 2.27 | - | 0.0007 |
|  |  | ΔP | 76 | 25.0 | 0.36 | - | <.0001 |
|  | trio^1/+^ | ∅ | 186 | 44.6 | 0.77 | 0.003 | - |
|  |  | WT | 91 | 8.8 | 0.09 | 0.5527 | <.0001 |
|  |  | Δ1Q | 82 | 80.5 | 2.28 | 1 | <.0001 |
|  |  | Δ1E | 88 | 17.0 | 0.26 | 1 | 0.0002 |
|  |  | Δ2E | 93 | 88.2 | 2.98 | 0.7905 | <.0001 |
|  |  | ΔP | 88 | 19.3 | 0.28 | 1 | 0.0005 |
|  | Sra-1^Df/+^ | ∅ | 60 | 8.3 | 0.10 | <.0001 | - |
|  |  | WT | 92 | 4.3 | 0.05 | 0.1055 | 1 |
|  |  | Δ1Q | 78 | 64.1 | 1.60 | 0.6832 | <.0001 |
