# Supplementary material for: The first quarter of the C-terminal domain of Abelson regulates the WAVE regulatory complex and Enabled in axon guidance
Source: Neural Dev. 2020 May 2;15:7. doi: 10.1186/s13064-020-00144-8 (PMC7196227; doi:10.1186/s13064-020-00144-8)
Supplement: Supplementary file 8 — Additional file 8 Table S6. Midline crossing over counts in Abl mutants expressing Abl transgenes with elav-Gal4, with heterozygous loss and overexpression of ena. [file 13064_2020_144_MOESM8_ESM.docx]

| **Genotype** | | | **n** | **% embryos with crossing over** | **Average crossing overs** | **p (to matched transgene in control)** | **p (to Abl^4/2^ within group)** |
| --- | --- | --- | --- | --- | --- | --- | --- |
| **Abl alleles** | **Other** | **Abl transgene** |  |  |  |  |  |
| Abl^4/+^ |  |  | 301 | 5.6 | 0.06 | - | <.0001 |
| Abl^4/2^ | controls | ∅ | 244 | 43.0 | 0.75 | - | - |
|  |  | WT | 156 | 9.6 | 0.12 | - | <.0001 |
|  |  | Δ1Q | 137 | 94.9 | 4.83 | - | <.0001 |
|  |  | Δ1E | 126 | 83.3 | 2.07 | - | <.0001 |
|  |  | Δ2E | 153 | 41.8 | 0.78 | - | 1 |
|  |  | ΔP | 147 | 26.5 | 0.40 | - | 0.0017 |
|  | ena^23/+^ | ∅ | 134 | 16.4 | 0.40 | 0.0028 | - |
|  |  | WT | 70 | 8.6 | 0.11 | 1 | 0.0139 |
|  |  | Δ1Q | 69 | 89.9 | 3.36 | 0.0139 | <.0001 |
|  |  | Δ1E | 68 | 79.4 | 3.00 | 0.0318 | <.0001 |
|  |  | Δ2E | 66 | 18.2 | 0.24 | 0.0005 | 0.7758 |
|  |  | ΔP | 47 | 21.3 | 0.47 | 1 | 1 |
|  | UAS-ena | ∅ | 187 | 98.9 | 6.71 | <.0001 | - |
|  |  | WT | 80 | 23.8 | 0.24 | 0.3236 | <.0001 |
|  |  | Δ1Q | 56 | 100.0 | 10.48 | <.0001 | 0.0001 |
|  |  | Δ1E | 97 | 48.5 | 0.84 | <.0001 | <.0001 |
|  |  | Δ2E | 62 | 100.0 | 4.34 | <.0001 | 0.0005 |
|  |  | ΔP | 74 | 78.4 | 1.99 | <.0001 | <.0001 |
